# Synthesis, Biophysical Properties, and Antitumor Activity of Antisense Oligonucleotides Conjugated with Anisamide

**DOI:** 10.3390/pharmaceutics15061645

**Published:** 2023-06-02

**Authors:** Zhe Zhang, Zuyi Chen, Cheng Li, Zhenyu Xiao, Yuan Luo, Xiaochen Pan, Liang Xu, Xuesong Feng

**Affiliations:** 1 School of Pharmacy, China Medical University, Shenyang 110122, China; 2 State Key Laboratory of Toxicology and Medical Countermeasures, Beijing Institute of Pharmacology and Toxicology, 27 Taiping Road, Beijing 100850, China; 3 Department of Orthopaedic Surgery, Beijing Jishuitan Hospital, Fourth Clinical College of Peking University, Beijing 100850, China; 4 Beijing Easyresearch Technology Limited, Beijing 100850, China

**Keywords:** oligonucleotide conjugation, anisamide, anti-enzymatic stability, cellular uptake, antitumor activity

## Abstract

Antisense oligonucleotides (ASONs) have proven potential for the treatment of various diseases. However, their limited bioavailability restricts their clinical application. New structures with improved enzyme resistance stability and efficient drug delivery are needed. In this work, we propose a novel category of ASONs bearing anisamide conjugation at phosphorothioate sites for oncotherapy. ASONs can be conjugated with the ligand anisamide very efficiently and flexibly in a solution. The conjugation sites and the ligand amount both influence anti-enzymatic stability and cellular uptake, resulting in changes in antitumor activity that are detectable by cytotoxicity assay. The conjugate with double anisamide (T6) was identified as the optimal conjugate, and its antitumor activity and the underlying mechanism were examined further in vitro and in vivo. This paper presents a new strategy for the design of nucleic acid-based therapeutics with improved drug delivery and biophysical and biological efficacy.

## 1. Introduction

Antisense oligonucleotides (ASONs) have been developed rapidly since the discovery in 1978 that they inhibit viral replication in cell culturing [1]. The mechanisms of ASONs can be divided into two broad groups: occupancy-mediated degradation (involving the cleavage of target RNAs); or occupancy only (involving steric interference) [2]. They are currently used for the treatment of metabolic and genetic diseases, cancer, the prevention of infectious diseases, etc. The advantages of ASONs include (i) their interaction with target genes by Watson–Crick base pairing, which provides specificity and affinity; and (ii) their ability to directly downregulate or upregulate gene expression, leading to the modulation of RNA metabolism [2,3].

ASON applications face multiple obstacles. A major issue is the degradation of naked ASONs by nucleases in plasma [4]. The half-life of naked ASONs is about 5 min, as determined in pharmacokinetic studies conducted with primates [5,6]. Numerous chemical modifications (e.g., of internucleoside linkages, deoxyribose/ribose, and nucleobases) have been developed to improve ASONs’ resistance to nuclease digestion [7]. ASON derivatization and bioconjugation have also been applied to confer resistance to serum enzyme degradation; for example, 5’ and 3’ spacer arms have been used to protect ASONs from exonuclease degradation [8].

Two broad strategies are currently applied for ASON delivery. In nanoscale delivery, ASONs are incorporated into nanocarriers for tissue distribution and cellular interactions. In molecular-scale delivery, ASONs are chemically modified, most commonly with targeting ligands, with the preservation of the molecular nature of the conjugates [9]. Such conjugates have received increasing interest recently because they enable selective delivery to specific cells and tissues via receptor-mediated mechanisms while avoiding the toxicity associated with many nanocarriers [9,10]. Another advantage of conjugates is that they are well-defined molecular entities, whereas nanoparticles (NPs) are heterogeneous. Moreover, some new technologies such as nonthermal plasma have been applied for the cellular transfection of oligonucleotide drugs [11].

Several small molecules show high degrees of affinity and selectivity for various receptors. The anisamide (AA) ligand binds to sigma receptors, which are membrane-bound proteins known to be overexpressed in various human tumors, such as melanoma, non-small-cell lung carcinoma, breast tumors originating from neural tissues, and prostate cancer [12,13].

Garg et al. [14] found that the addition of AA to chitosan/poly (ethylene glycol) NPs significantly increased the NPs’ affinity and thus, their ability to successfully deliver drugs to human lung carcinoma cells in vitro.

**Figure 1:**
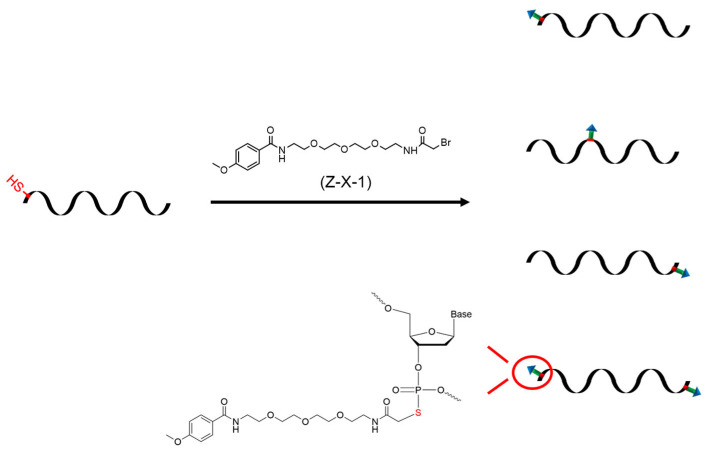
The design of ASONs for AA conjugation.

In this work, a novel category of ASONs with AA conjugation for oncotherapy is proposed (Figure 1). Conjugation can be achieved between an acetyl bromide-modified AA derivate and an ASON with phosphorothioate (PS) modification to improve the anti-enzymatic stability and the cellular uptake capability, with the potential to enhance antitumor activity.

To provide evidence of the validity of the approach, the 20-mer ASON GTI-2040 was utilized as the lead molecule in this study. GTI-2040, which was developed by Loris Therapeutics (Toronto, ON, Canada) for the treatment of cancer, is designed to be complementary to a coding region in the mRNA of human ribonucleotide reductase’s (RR) R2 small subunit component [15,16]. Five conjugates with different structures were synthesized, and their biophysical properties and tumor cell inhibition capacities were characterized. The antineoplastic effects of the optimal molecule were examined further in vitro and in vivo.

## 2. Materials and Methods

### 2.1. Materials

Dimethyl sulfoxide (DMSO), triethylamine, pyridine, ammonia, dichloromethane, methanol, petroleum ether, ethyl acetate, sodium carbonate, and magnesium sulfate were obtained from China National Medicines Corporation, Ltd., Beijing, China. Sodium chloride was provided by Xilong Science Co., Ltd., Guanzhou, China. Column chromatography silica gel was provided by Qingdao Marine Chemical Co., Ltd., Qingdao, China. Pure water was obtained from WaHaHa. Hydrochloric acid was provided by Beijing Tongguang Fine Chemical Co., Ltd., Beijing, China. Tetraethylene glycol (CAS: R4XRE0RD), bis(t-butyloxycarbonyl) amine (CAS: GC190009), p-methoxybenzoyl chloride (CAS: 97LERTHD), anhydrous dichloromethane (CAS: BMROAEFH), bromoacetyl bromide (CAS: 9MTRRE3X), and 4-toluenesulfonyl chloride were obtained from Energy Chemical (Shanghai, China). Cesium carbonate (CAS: K1624005) was purchased from Aladdin.

### 2.2. Synthesis of Z-X-1 and Z-X-2 Compounds

**Scheme 1:**
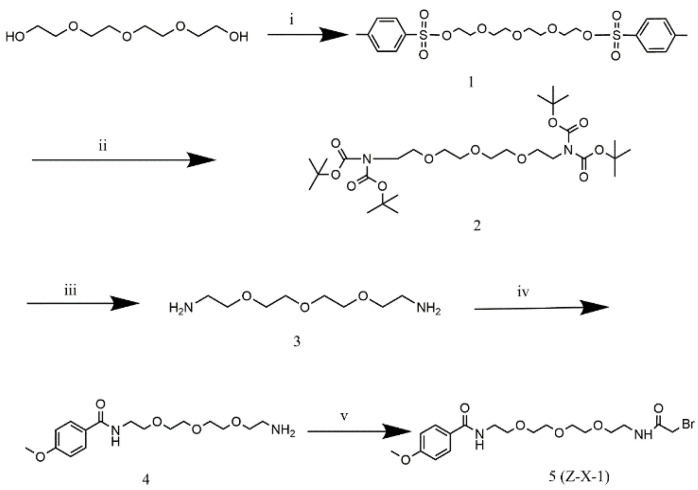
The synthetic route of the Z-X-1 compound. (**i**) Toluenesulfonyl chloride, CHCl_2_, room temperature, overnight; (**ii**) bis(tert-butoxycarbonyl) amine, cesium carbonate, SOCl_2_, 80 °C, 2 h; (**iii**) hydrochloric acid/ethyl acetate, room temperature, overnight; (**iv**) p-methoxybenzoyl chloride, CHCl_2_, 0 °C to room temperature, 3 h; (**v**) bromoacetyl bromide, Et_3_N, CHCl_2_, room temperature, overnight.

**Scheme 2:**
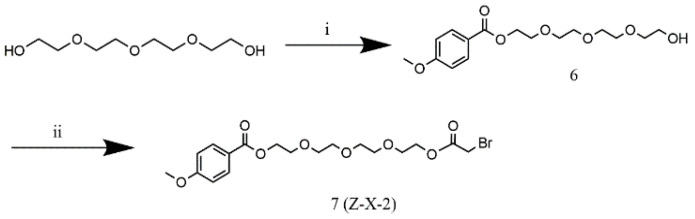
The synthetic route of the Z-X-2 compound. (**i**) Toluenesulfonyl chloride, CHCl_2_, room temperature, overnight; (**ii**) bromoacetyl bromide, Et_3_N, CHCl_2_, room temperature, overnight.

The synthesis of Z-X-1 and Z-X-2 compounds is shown in Scheme 1 and Scheme 2 (details are provided in the Supporting Materials). All new compounds were fully characterized by mass spectrometry (MS; AB Sciex API4000, SCIEX, Framingham, MA, USA) and ^1^H nuclear magnetic resonance (NMR; INOVA, Agilent, Santa Clara, CA, USA) imaging.

### 2.3. Oligonucleotide Synthesis and Characterization

The oligonucleotides used in this study were procured from TSINGKE Co., Ltd. (Beijing, China). Their molecular weights were determined using matrix-assisted laser desorption ionization–time-of-flight mass spectrometry (MALDI-TOF MS; KRATOS Analytical, Shimadzu Group, Kyoto, Japan). The matrix used 2′,4′,6′-trihydroxyacetophenone, and the acceleration voltage was set to 20 kV.

### 2.4. Preparation of AA-ASON Conjugates (T2–T6)

The Z-X-1 and Z-X-2 compounds were dissolved in d_6_-DMSO (8 mM) and mixed with ASONs Z1, Z2, Z3, and Z4 (400 Mm; Table S1), respectively, in sterilized water. The molar ratio between the ASONs and the first compound was 1:10. The mixtures were shaken gently at 50 °C for 4 h to complete the reactions. Conjugates (T2–T6) were purified using reverse-phase high-performance liquid chromatography (HPLC). The purification process involved the use of a chromatography column with buffer A (containing 0.1 M acetic acid/triethylamine mixed with 5% acetonitrile) and buffer B (acetonitrile), with a gradient of B in A, which increased from 5% to 70% over a period of 20 min at a flow rate of 1 mL/min. Subsequently, desalting was performed using SEP-PAK cartridges (Oasis MCX, C18; Waters). The cartridges were washed repeatedly in sterilized double-distilled water and then lyophilized before being stored at −18 °C.

### 2.5. Assessment of ASONs’ Resistance to Degradation in Serum- and DNase I–Containing Buffers

To assess the resistance of the ASONs against nuclease degradation, each oligodeoxynucleotide (4.7 μmol) was introduced to 10 μL of culture medium infused with either 40% fetal bovine serum (FBS) or DNase I enzyme. Subsequently, 3 μL of formamide was added to each sample as a loading buffer before performing a 20% polyacrylamide gel electrophoresis (PAGE) assay. The results were analyzed using ImageJ software (developed by Broken Symmetry Software; version number: V1.8.0.112).

### 2.6. Target mRNA Binding Assay

T1 (4.7 μmol) and T6 (4.7 μmol) were tested with simulated target mRNA areas (4.7 μmol) in a solution of phosphate-buffered saline (PBS), which had 100 mM NaCl and 10 mM MgCl_2_ (1 mL). The absorbance changes at 260 nm caused by the temperature escalation from 20 °C to 90 °C (at 1 °C/min) were observed using a Cary-100 Bio UV–visible spectrophotometer (Varian in Palo Alto, CA, USA). The melting temperatures were calculated based on the first-derivative plots of absorbance against temperature.

### 2.7. In Vitro Studies

#### 2.7.1. Cell Culture

The in vitro antitumor activity of ASONs was assessed using MCF-7 cells (National Infrastructure of Cell Line Resources, Beijing, China). These cells were cultured in Roswell Park Memorial Institute 1640 medium, supplemented with 10% FBS, and maintained at 37 °C and 5% CO_2_ until subconfluence (75–85%).

#### 2.7.2. Assessment of Cellular Uptake

MCF-7 cells were seeded in glass-bottomed dishes at densities of either 6 or 8 × 10^4^ cells/mL and allowed to adhere for a period of 24 h. Fluorescein amidite (FAM)-labeled ASONs (2 μM) were added and incubated for 4 h. The cells were then fixed with 4% paraformaldehyde and washed three times with 2 mL PBS, and 2 mL of the dihydrochloride (DAPI, 10 μg/mL) regent was added. Confocal scanning laser microscopy (CLSM) was used to evaluate cellular uptake. With a confocal microscope (SUNNY CSIM 110), lasers at 405 and 488 nm were used for the fluorescence excitation of DAPI and FAM, respectively. 

MCF-7 cells were seeded onto 12-well plates at a density of 3 × 10^5^ cells/well and allowed to attach and grow for a period of 24 h. FAM-labeled ASONs were then added to each well and incubated for 4 h. The cells were washed three times with 300 μL PBS and then 300 μL 4% paraformaldehyde was added, followed immediately by flow cytometric analysis (FACSCalibur; BD Biosciences, Ramsey, MN, USA). The fluorescence intensity values for 10,000 events/sample were analyzed at an excitation/emission ratio of 494/522 nm. Relative mean fluorescence intensities (MFIs) were determined by dividing the fluorescence intensities of the different AA-ASONs by that of the PBS group. 

#### 2.7.3. Assessment of Cytotoxicity Study

Linear polyethylenimine (PEI) reagent (cat. #40816ES03; Shanghai YEASEN Biotechnology, Shanghai, China) was used for transfection. It was added with prepared ASONs (transfection, 0.6 μM; direct administration, 2 μM) to MCF-7 cells and incubated for 24 h. Then, the MCF-7 cell proliferation rate was determined using the cell counting kit 8 (CCK8) method. A total of 10 μL of CCK-8 reagent (cat. #40203ES92, Shanghai YEASEN Biotechnology, Shanghai, China) was added to each well and then cultured for 40 min. All experiments were performed in triplicate. The absorbance was measured at 450 nm using a microplate reader (E-max; Molecular Devices, Sunnyvale, CA, USA), with wells without cells serving as blanks. Cell proliferation was evaluated based on the absorbance values.

#### 2.7.4. Assessment of the Mechanism of Cellular Uptake

MCF-7 cells were seeded onto 12-well plates at a density of 3 × 10^5^ cells/well and allowed to attach and grow for a period of 24 h. Three pharmaceutical endocytosis inhibitors (amiloride, chlorpromazine (CPZ), and methyl-β-cyclodextrin(MβCD)) and haloperidol pretreated the cells for 30 min. FAM-labeled ASONs were then added to each well and incubated for 4 h. The cells were washed three times with 300 μL PBS, and then 300 μL 4% paraformaldehyde was added, followed immediately by flow cytometric analysis (FACSCalibur; BD Biosciences, Ramsey, MN, USA). The fluorescence intensity values for 10,000 events/sample were analyzed at an excitation/emission ratio of 494/522 nm.

### 2.8. Cell Apoptosis Experiment

To provide additional validation of T6′s antitumor properties, the apoptosis of tumor cells was measured using our previously reported method [17]. In brief, the ASONs were incubated with MCF-7 cells for 24 h. After this incubation period, 1 × 10^5^ cells were collected and suspended in 100 μL of binding buffer containing annexin V–fluorescein isothiocyanate (FITC) and propidium iodide (PI) for a 15 min period. The samples were subjected to flow cytometry (FACSCalibur; BD Biosciences).

### 2.9. Reverse-Transcriptase Quantitative Polymerase Chain Reaction Assay

A reverse-transcriptase quantitative polymerase chain reaction (RT-qPCR) assay was conducted according to our previously reported protocol [18]. Briefly, MCF-7 cells were seeded in six-well plates at a density of 5 × 10^4^ cells/well. After 24 h of incubation, T1 or T6 (0.2 μM) was added, and the cells were further incubated for 48 h. Total RNA was isolated using 1 mL of TRIzol Reagent (Invitrogen, Carlsbad, CA, USA), and cDNA was synthesized using the PrimeScript RT reagent kit with gDNA eraser (code DRR047A; TaKaRa). The RT-qPCR was performed using SYBR Premix Ex Taq II reagent (code DRR081A; TaKaRa) with the standard cycling program (40 cycles of 95 °C for 30 s and 60 °C for 5 s), and melting curves were generated by a temperature increase from 65 °C to 95 °C at a rate of 0.5 °C/5 s using a CFX96 real-time PCR detection system (Bio-Rad Laboratories Ltd., Hercules, CA, USA). The forward and reverse primer sequences for RRM2 are shown in Table 1. The calculations were performed using the CFX Manage software (version 1.0; Bio-Rad Laboratories Ltd.). The results are expressed as fold changes in expression [2^(−ΔΔCt)^] on a linear scale.

### 2.10. In Vivo Studies

Nude BALB/c mice (aged 6–7 weeks, weighing 20 ± 2 g; Beijing Keyu Animal Breeding Center, Beijing, China) were used for the in vivo assessment of the ASONs’ antitumor activity. A suspension of MCF-7 cells (1 × 10^5^) in 100 μL of PBS was subcutaneously inoculated into the right flank of the animals. The treatments began after the average tumor volume had reached about 80 mm^3^. Fifteen animals with tumors were randomly divided into three groups: PBS-, T1-, and T6-treated groups. The treatments (0.1 mL (0.3 mmol/L)) were administered by intra-tumor injection daily for 14 days. The mice were sacrificed following the final administration of treatment. The tumor volumes ((length × width × height)/2) were measured with a caliper. Mean tumor volume was calculated by dividing the sum of tumor volumes in a group by the number of mice in that group. Tumor samples were obtained, fixed overnight in 10% formalin, and subsequently embedded in paraffin before being cut to a thickness of 4 μm. These sections were then deparaffinized, rehydrated, and subjected to Hematoxylin and Eosin (H&E) staining.

### 2.11. Statistical Analysis

All data are presented as means ± standard deviations. Significant differences were identified using the two-tailed Student’s *t* test. The significance level was set to *p* < 0.05.

## 3. Results and Discussion

### 3.1. Z-X-1 and Z-X-2 Compounds

The Z-X-1 compound was synthesized with a typical AA region connected to an active acetyl bromide group by a flexible oxyalkyl chain as a ligand for conjugation with the ASONs. The Z-X-2 compound was synthesized by replacing the nitrogen atom in AA with an oxygen atom as a negative control. The MS, ^1^H NMR, and ^13^C NMR results confirmed that the desired structures and intermediates had been obtained (Figures S1–S21).

### 3.2. ASON Conjugation

**Figure 2:**
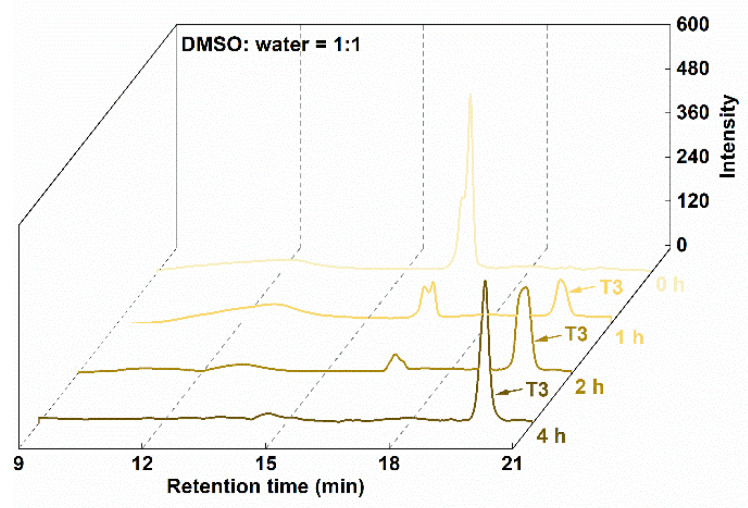
The HPLC spectra of T3.

The acetyl bromide group’s ability to react with any PS group in the solution phase facilitates the design of ASON–AA conjugates with different ligand sites and amounts. ASONs with different PS modifications (at the 3′ and/or 5′ terminal and in the middle) were used for conjugation (Table 2). The confirmation of the ASON molecular weights by MALDI-TOF MS and HPLC is shown in Figures S26–S36. Conjugation was attempted under several reaction conditions with different components and phase solvent ratios; the synthesis of T3, as an example, is detailed in Table 3. The conditions affected the reaction times and yields. The best condition was identified as the reaction for 4 h in a d_6_-DMSO/aqueous solution, which provided a high yield (98.9%), as confirmed by HPLC analysis (Figure 2). In addition, compared to traditional solid-phase synthesis in other research, our conjugation was found with high efficiency and site flexibility, which provides convenience for constructing more DNA-based conjugates [9].

### 3.3. Anti-Serum and Anti-Enzymolysis Stability

Serum is known to contain a significant number of nucleases that can easily degrade naked DNA. DNase I is an endonuclease that can cleave both single- and double-stranded DNA nonspecifically [19].

**Figure 3:**
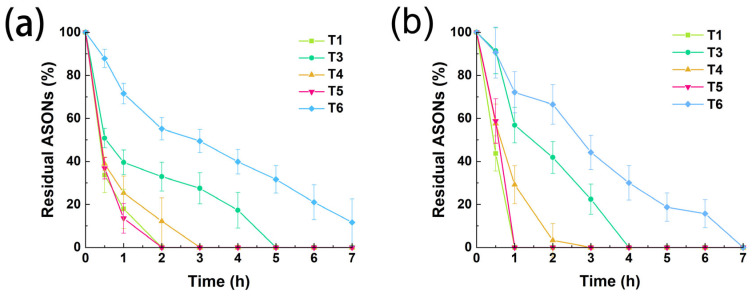
The stability of T1 and T3–T6 against enzymatic degradation in (**a**) serum- and (**b**) DNase I-containing media.

In serum, native linear T1 degraded completely within 2 h (Figure 3a). Among the single AA–ASON conjugates, the intermediately modified T5 degraded most rapidly. T3, with AA conjugation at the 3’ end, was more stable than T4, with AA conjugation at the 5’ end. This result can be explained by the mediation of degradation primarily by a 3’ exonuclease and the ability of AA coupling to near-3’-end ASONs to protect against serum 3’ exonuclease-mediated degradation [20,21]. T6, with double-end protection, was the most stable, with >10% remaining after 7 h.

The degradation trends of T1 and T3–T6 in DNase I-containing medium were similar to those in serum, with T6 being the most stable ASON (Figure 3b). These results indicate that double-ended AA–ASON conjugation greatly improves stability against enzymatic degradation. The resistance to enzymatic degradation was comparable to that achieved in our previous research, which utilized spatial conformation modification at the 3′ and 5′ ends [17].

### 3.4. In Vitro Cellular Uptake

**Figure 4:**
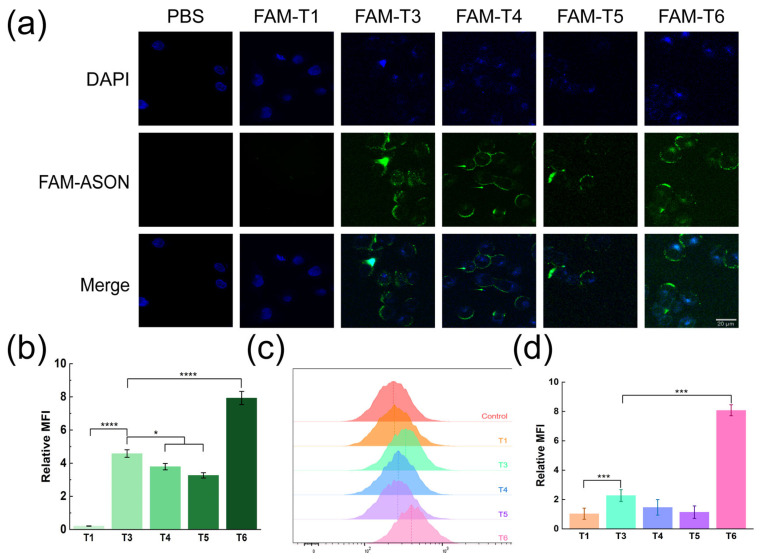
The cellular uptake of the ASON conjugates. (**a**) Confocal images; (**b**) the relative MFIs of its confocal images; (**c**) flow cytometric analyses of treated MCF-7 cells (nuclei are labeled with DAPI (blue) and ASONs are labeled with FAM (green); scale bar = 40 μm). (**d**) Relative MFIs of treated MCF-7 cells. * *p* < 0.05 vs. T3. *** *p* < 0.001 vs. T3. **** *p* < 0.0001 vs. T3. The data are presented as means ± standard deviations (*n* = 3).

CLSM revealed almost no fluorescence intensity in the T1 group and more pronounced intensities in the other groups (Figure 4a,b). Among the single-AA ASONs, T3 had stronger fluorescence intensity than T4 and T5 did. T6 showed the greatest fluorescence intensity, not only around the cell membranes, but also partly penetrating the nuclei. These results indicate that ASONs conjugated with AA groups entered cells more efficiently than did the native ASON and that double-ended AA conjugation yielded the best results. They also indicate that the AA amount and positions affect the cellular uptake ability. The results of flow cytometry were in agreement with those of CLSM (Figure 4c). The MFIs of T3–T5 were greater than that of T1. The MFI of T6 was 7.7 times greater than that of T1 (*p* < 0.001) (Figure 4d). These results indicated that AA conjugation enhanced the cellar uptake of ASONs. Compared with other studies of nanocarriers modified with AA [22], the ASON–AA conjugate has potential to avoid toxicities often associated with nanocarriers [9]. The Z-X-2 compound was selected as the negative control ligand and used to synthesize an anisyl ester–ASON conjugation (T2) because the nitrogen atom is an essential pharmacophore in the binding of phenyl alkyl molecules to α receptors [23]. The cellular uptake abilities of T2 and T1 did not differ significantly, with almost no fluorescence observed in both cases (Figure S37).

### 3.5. In Vitro Cytotoxicity

**Figure 5:**
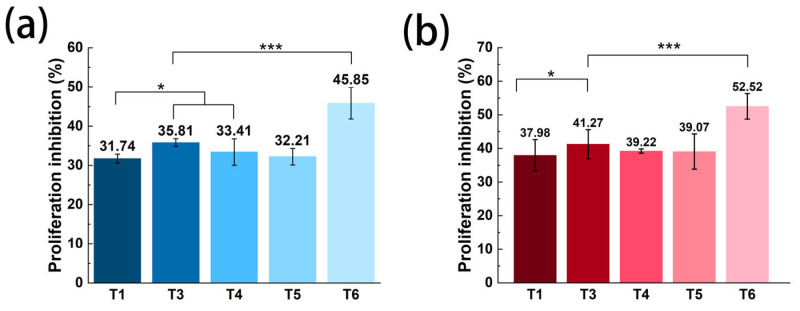
The cytotoxicity of T1 and T3–T6 with PEI transfection and an ASON concentration of 0.2 μM (**a**) and with direct administration with an ASON concentration of 0.6 μM (**b**). * *p* < 0.05, *** *p* < 0.001 vs. T3. The data are presented as means ± standard deviations (*n* = 4).

The linear PEI is a commonly used transfection tool that can deliver molecules into cells based on the so-called ‘proton sponge mechanism. Many studies have adopted this method to investigate the effect of antisense therapy, and our study also used PEI for transfection at the beginning of this experiment. T1, T3, and T4–T6 were subjected to cytotoxicity assays, and the inhibition of tumor cell proliferation was assessed using the CCK8 method. Linear PEI was used as the nucleic acid transfection reagent [24]. With PEI transfection, the T6 group showed greater inhibition of tumor cell proliferation than did groups T3–T5 (all *p* < 0.001) and the T1 group (Figure 5a).

In addition, considering that the marketed ASONs do not use delivery vectors and their structural modifications promote cellular uptake, we also attempted to determine whether direct administration of T3–T6 is effective. ASONs can enter cells via endocytosis, and no ASON drug currently on the market employs a nanocarrier; thus, the improvement of ASON cellular uptake is of importance. In the direct-administration (i.e., without transfection) cytotoxicity assays, the T6 group still showed the best proliferation inhibition (*p* < 0.001 vs. T3–T5; Figure 5b). This inhibition ability may be due to T6′s double-ended protective structure and improved cellular uptake ability.

Taken together, the results led to the selection of T6 as the optimal molecule for further research.

### 3.6. Binding Affinity of T6 to Target mRNA

Melting temperatures during incubation with a simulated target RNA region did not differ significantly between the T6 and T1 groups (Table 4), suggesting that the conjugated structure of T6 did not affect its binding to the target mRNA.

### 3.7. Mechanism of Cellular Uptake

**Figure 6:**
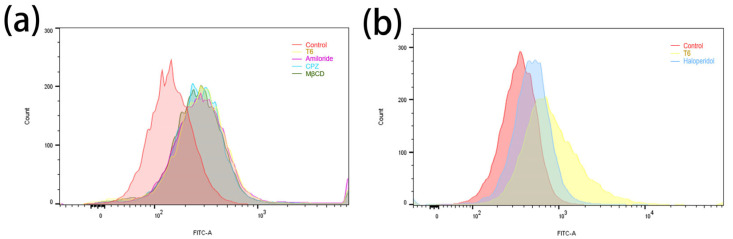
Cellular uptake pathways of T6 with untreated MCF-7 cells and cells pretreated with three pharmaceutical endocytosis inhibitors (amiloride, CPZ, and MβCD; (**a**) and a sigma-receptor antagonist (haloperidol) (**b**).

To investigate the cellular uptake mechanism of T6, three pharmaceutical endocytosis inhibitors (amiloride, chlorpromazine (CPZ), and methyl-β-cyclodextrin (MβCD)) were used to block the established pathways, and their contributions were assessed using flow cytometry. Amiloride inhibits caveolin-mediated macropinocytosis, CPZ inhibits clathrin-mediated endocytosis, and MβCD inhibits caveolin-mediated endocytosis. Treatment with the three inhibitors did not significantly reduce the cellular uptake ability of T6 (Figure 6a). However, this ability was inhibited significantly when the MCF-7 cells were pretreated with haloperidol (a sigma-receptor antagonist; Figure 6b) [12,22]. These results suggest that the enhanced cellular uptake of T6 was mediated primarily by sigma receptors. To our knowledge, the precise molecular structure and mechanism of sigma-1R interacting with AA still need to be fully elucidated [25]. 

### 3.8. In Vitro Antitumor Mechanism of T6

**Figure 7:**
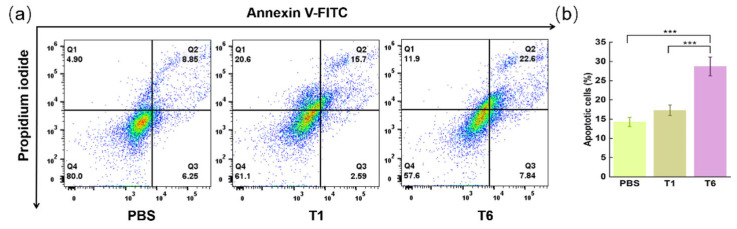
(**a**) The apoptosis of MCF-7 cells treated with PBS, T1, and T6 detected by flow cytometry. (**b**) Percentages of apoptotic MCF-7 cells. *** *p* < 0.001 vs. T6. The data are presented as means ± standard deviations (*n* = 3).

To further confirm the antitumor effect of T6, the apoptosis of tumor cells was measured by flow cytometry. Annexin V–FITC^−^/PI^−^ cells were identified as living (Figure 7a, lower left quadrant), annexin V–FITC^+^/PI^−^ cells were identified to be in the early stage of apoptosis (Figure 7a, upper left quadrant), annexin V–FITC^+^/PI^+^ cells were identified to be in the late stage of apoptosis (Figure 7a, upper right quadrant), and annexin V–FITC^−^/PI^+^ cells were identified as necrotic (Figure 7a, upper left quadrant). The apoptosis-promoting effect was reflected by the proportions of fluorescence scatter intensity in the Q2 and Q4 regions. The T6-treated group showed a significant apoptotic effect, with an apoptotic cell population of 30.44% relative to those of the PBS-treated group (15.10%) and the T1-treated group (18.29%; both *p* < 0.001; Figure 7).

### 3.9. RT-qPCR Results

**Figure 8:**
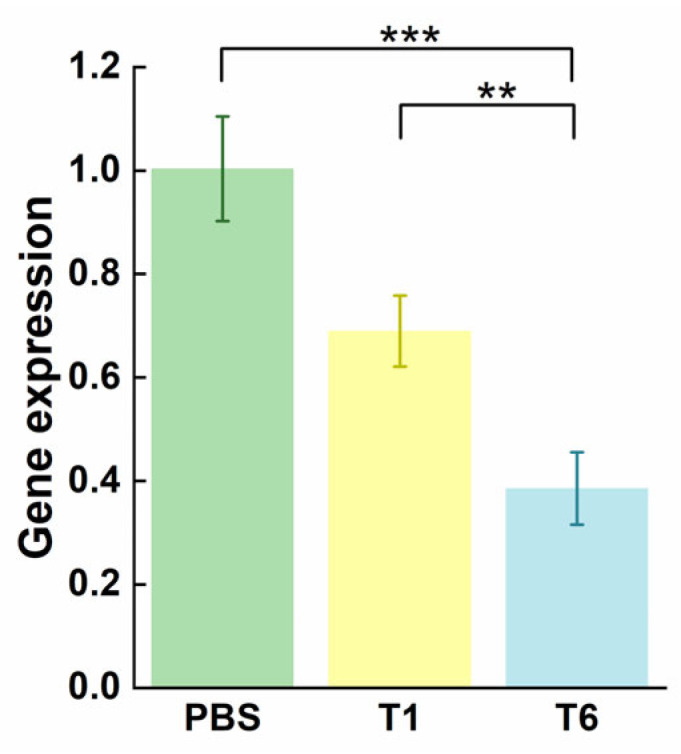
Relative expression of RRM2 mRNA after MCF-7 cell treatment with PBS, 2 μM T1, or 2 μM T6. ** *p* < 0.01, *** *p* < 0.001 vs. T6. The data are presented as means ± standard deviations (*n* = 3).

RR is composed of large (R1) and small (R2) subunits. R2 is encoded by the RRM2 gene on chromosome 12 and plays a role as a tumor promoter that can enhance the transformation and malignancy potential of cells in cooperation with various oncogenes [26,27]. A specific ASON, GTI-2040, has been demonstrated to directly and selectively inhibit the complementary binding of RRM2 mRNA to the coding region. To investigate the effect of T6 treatment on RRM2 mRNA expression, an RT-qPCR assay was performed to compare the relative expression of RRM2 mRNA levels in the study groups. The results revealed that T6 significantly suppressed the mRNA expression of RRM2 in MCF-7 cells compared to that in the T1- and PBS-treated groups (both *p* < 0.001; Figure 8). These results suggest that T6 significantly suppressed RRM2 mRNA expression.

### 3.10. In Vivo Antitumor Capacity of T6

**Figure 9:**
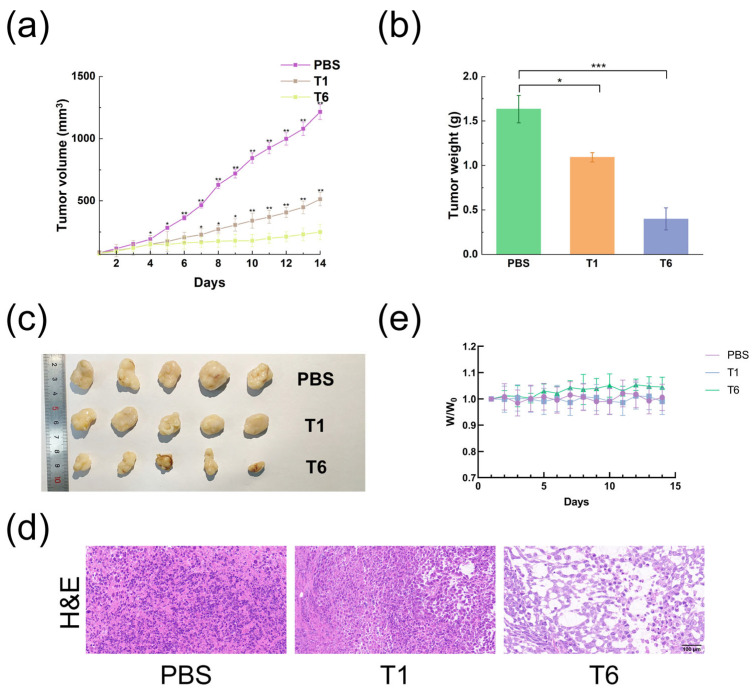
In vivo antitumor activity of T6, T1, and PBS. (**a**) The growth of MCF-7 tumor xenografts in mice under the different treatments. * *p* < 0.05, ** *p* < 0.01 vs. T6. The data are presented as means ± standard deviations (*n* = 5). (**b**) Post-treatment tumor weights. * *p* < 0.05, *** *p* < 0.001 vs. PBS. The data are presented as means ± standard deviations (*n* = 5). (**c**) Photograph of tumors isolated from mice after different treatments. (**d**) H&E staining of MCF-7 tumor tissues after 14 days of treatment. (**e**) Body weights of MCF-7 tumor-bearing mice under treatment.

We evaluated the in vivo antitumor activity of PBS, T1, and T6 in a nude mouse model with human breast cancer (MCF-7) cells. Intratumoral injections were administered daily. We assessed the therapeutic efficacy of the treatment regimens by measuring tumor growth and weight. Tumors formed and grew rapidly in mice treated with PBS. T1 had a moderate tumor inhibition effect, and T6 significantly slowed tumor growth (Figure 9a–c).

Histological analysis of H&E-stained samples revealed no obvious malignant necrosis in the PBS and T1 groups but massive cell remission and a high rate of apoptosis in the T6 group (Figure 9d), confirming that T6 efficiently suppressed tumor growth. These results are likely attributable to the increased resistance of T6 to nuclease degradation and cellular uptake, observed in vivo. No significant change in mouse body weight was observed during treatment in any group (Figure 9f), demonstrating the biocompatibility of T6.

## 4. Conclusions

In this work, ASONs with AA conjugation at desired PS sites were synthesized for oncotherapy. Both the conjugation sites and the ligand amount influence the physical and chemical properties. An optimal conjugate, T6 (with double end AA-conjugation), showed significant improvements in stability against enzymatic hydrolysis, cellular uptake, and biological effects. This paper presents a new strategy for the design of nucleic acid-based therapeutics with improved drug delivery and biophysical and biological efficacy. The potential applications of ASONs combined with cutting-edge techniques, such as nonthermal plasma, could be carried out in future research.

## Figures and Tables

**Table 1:** RT-qPCR primers.

Gene	Sequence
β-actin	Forward: 5′-CGAGCGCGGCTACAGCTT-3′
	Reverse: 5′-CCTTAATGTCACGCACGATT-3′
RRM2	Forward: 5′-ATGAAAACTTGGTGGAGCGATT-3′
	Reverse: 5′-TGGCAATTTGGAAGCCATAGA-3′

**Table 2:** ASON sequences and molecular weights.

No.	Sequence (5′-3′)	Molecular Weight (Estimated)	Molecular Weight (Actual)
T1	GGCTAAATCGCTCCACCAAG	6071.0	6071.0
T2	GGCTAAATCGCTCCACCAA #G	6453.3	6453.1
T3	GGCTAAATCGCTCCACCAA *G	6455.0	6454.7
T4	G*GCTAAATCGCTCCACCAAG	6455.0	6453.3
T5	GGCTAAATCG*CTCCACCAAG	6455.0	6454.7
T6	G*GCTAAATCGCTCCACCAA *G	6836.8	6836.1

# PS sites connecting with the Z-X-2 compound; * PS sites connecting with the Z-X-1 compound.

**Table 3:** Optimization of coupling conditions.

Composition of Coupling Solvent		Coupling Solvent Ratio		Reaction Time (h)	Yield (%)
Organic Phase(A)	Aqueous(B)	A	B		
d_6_-DMSO	Sterile water	1	1	4	98.9
d_6_-DMSO	Sterile water	1	4	5	95
Acetonitrile	Sterile water	1	1	12	0
Acetonitrile	Sterile water	1	4	12	0
Acetonitrile	PBS	1	1	12	0
Acetonitrile	PBS	1	4	12	0
Acetonitrile	PBS + 100 mM NaCl	1	1	12	0
Acetonitrile	PBS + 100 mM NaCl	1	4	12	0
Methanol	Sterile water	1	1	6	70.1
Methanol	Sterile water	1	4	6	64.3
Methanol	PBS	1	1	12	27.2
Methanol	PBS	1	4	12	25.8
Methanol	PBS + 100 mM NaCl	1	1	12	28.9
Methanol	PBS + 100 mM NaCl	1	4	12	25.3

**Table 4:** Melting temperatures of T1 and T6 binding to the target mRNA ^#^.

	T1	T6
T_m_	67.54 ± 0.26	68.56 ± 1.51
*p* value		0.30

^#^ The 5′ to 3′ sequence was CUUGGAGCGUUUAGCC.

## Data Availability

Not applicable.

## Supplementary Materials

The following supporting information can be downloaded at https://www.mdpi.com/article/10.3390/pharmaceutics15061645/s1: Figures S1–S21, 1H NMR, 13C NMR and MS spectra of the Z-X-1 and Z-X-2 compounds; Table S1, Z1–Z4 sequences and molecular weights; Figures S22–S25, MALDI-TOF-MS spectra of Z1–Z4; Figures S26–S36, MALDI-TOF-MS and HPLC spectra of T1–T6; Figure S37, Cellular uptake ability of T1–T3. (a) Confocal images and (b) flow cytometry of MCF-7 cells treated with PBS and T1–T3. (c) Relative MFIs of treated MCF-7 cells.
