# Supplementary material for: Synthesis, Biophysical Properties, and Antitumor Activity of Antisense Oligonucleotides Conjugated with Anisamide
Source: Pharmaceutics. 2023 Jun 2;15(6):1645. doi: 10.3390/pharmaceutics15061645 (PMC10302603; doi:10.3390/pharmaceutics15061645)
Supplement: Supplementary file 1 [file pharmaceutics-15-01645-s001.zip › pharmaceutics-2366091-supplementary.pdf]

pharmaceutics

MDPI

Synthesis, biophysical characterization, and antitumor activity of a tisense oligonucleotides with anisamide conjugation

Zhe Zhang[1,2]†, Zuyi Chen[1,2]†, Zhenyu Xiao[2], Yuan Luo[2], Xiaochen Pan[3], Liang Xu[2,]* and Xuesong Feng[1]

1. Synthesis of the Z-X-1 and Z-X-2 compounds

Compound 1: [(oxybis(ethane-2,1-diyl) bis(oxy)] bis(ethane-2,1-diyl) bis(4-methylbenzenesulfonate)

Tetraethylene glycol (1 g, 5.15 mmol) was dissolved in dichloromethane. 4-Toluenesulfonyl chloride (2.95 g, 15.45 mmol) dissolved in pyridine was dripped slowly into this solution and stirred overnight at room temperature. The progress of the raw material reaction was monitored by thin-layer chromatography (TLC). The reaction solution was then diluted with distilled water, and the water layer was extracted with dichloromethane. The organic layer was washed successively with hydrochloric acid and saturated NaCl solution, then dried with $MgSO_4$ and concentrated. The residue was purified with flash chromatography (petroleum ether:ethyl acetate = 1:1) to yield a colorless, transparent oily substance (2.32 g, 89.6%). ^{1}H NMR (600 MHz, DMSO-d_6) δ 7.81–7.75 (m, 4H), 7.50–7.45 (m, 4H), 4.13–4.08 (m, 4H), 3.59–3.54 (m, 4H), 3.46–3.39 (m, 8H), 2.42 (s, 4H). MS m/z: $[M+H]^-$:503.14. ^{13}C NMR (151 MHz, DMSO-d_6) δ 145.37, 132.87, 130.60, 128.09, 70.45, 70.12 (d, J = 4.6 Hz), 68.34, 21.56.

Compound 2: di-tert-butyl {[(oxybis(ethane-2,1-diyl)] bis(oxy)} bis(ethane-2,1-diyl) bis[(tert-butoxycarbonyl) carbamate]

Bis (tert-butoxycarbonyl) amine (0.87 g, 3.98 mmol) and cesium carbonate (1.3 g, 3.98 mmol) were dissolved in dimethyl sulfoxide. Compound 1 (1 g, 1.99 mmol) was then added. The mixture was stirred at 80°C for 2 h. The progress of the raw material reaction was monitored by TLC. The reaction solution was then diluted with distilled water, and the compound 2 was extracted with ethyl acetate and washed with saturated NaCl solution. The organic layer was dried with anhydrous $MgSO_4$ and concentrated. The residue was purified with flash chromatography (petroleum ether: ethyl acetate = 5:1) to yield a colorless, transparent oily substance (0.32 g, 27.2%). ^{1}H NMR (600 MHz, DMSO-d6) δ 3.64 (t, J = 5.9 Hz, 4H), 3.50–3.46 (m, 12H), 1.43 (s, 36H). MS m/z $[M+Na]^-$: 615.36. ^{13}C NMR (151 MHz, DMSO-d_6) δ 152.60, 82.16, 70.28, 70.02, 68.85, 45.41, 28.04.

Compound 3: 2,2′-[(oxybis(ethane-2,1-diyl) bis(oxy)] bis(ethan-1-amine)

Compound 2 (0.1 g, 0.169 mmol) was added to hydrochloric acid/ethyl acetate (10 ml) and stirred overnight at room temperature. The solvent was then evaporated. The progress of the raw material reaction was monitored by TLC. The reaction solution was concentrated by rotatory evaporation to yield a yellow oily product (0.017 g, 52.4%). ^{1}H NMR (600 MHz, DMSO-d_6) δ 8.37 (t, J = 5.6 Hz, 1H), 7.84–7.81 (m, 2H), 7.00–6.97 (m, 2H), 3.80 (s, 3H), 3.60–3.49 (m, 12H), 3.40 (q, J = 6.0 Hz, 3H), 2.96 (t, J = 5.3 Hz, 2H), 2.00 (s, 2H). MS m/z [M+Na]$^-$: 327.19. ^{13}C NMR (151 MHz, DMSO-d_6) δ 73.22, 70.16 (d, J = 32.2 Hz), 41.68.

Compound 4: N-(2-{2-[2-(2-aminoethoxy)ethoxy]ethoxy}ethyl)-4-methoxybenzamide

Compound 3 (0.5 g, 2.6 mmol) and pyridine were dissolved in dried dichloromethane. Under nitrogen protection in an ice bath, p-methoxybenzoyl chloride (0.15 g, 0.87 mmol) was dissolved in dried dichloromethane and then added to the solution drop by drop, followed by stirring in an ice bath for 1 h and at room temperature for 3 h. The progress of the raw material reaction was monitored by TLC. The mixture was partitioned between saturated NaCl solution and CH_2Cl_2. The organic layer was dried with anhydrous $MgSO_4$ and concentrated. The residue was purified with flash chromatography (dichloromethane:methanol = 8:1) to yield a white crystalline substance (0.08 g, 29.5%). ^{1}H NMR (600 MHz, DMSO-d_6) δ 8.37 (t, J = 5.6 Hz, 1H), 7.84–7.81 (m, 2H), 7.00–6.97 (m, 2H), 3.80 (s, 3H), 3.60–3.49 (m, 12H), 3.40 (q, J = 6.0 Hz, 3H), 2.96 (t, J = 5.3 Hz, 2H), 2.00 (s, 2H). MS m/z [M+Na]$^-$: 327.19. ^{13}C NMR (151 MHz, DMSO-d_6) δ 166.21, 161.95, 129.44, 127.12, 113.90, 73.53, 70.24, 70.22, 70.10, 70.02, 69.49, 55.79, 49.06, 41.82.

Compound 5: N-(1-bromo-2-oxo-6,9,12-trioxa-3-azatetradecan-14-yl)-4-methoxybenzamide (Z-X-2)

Compound 4 (0.2 g, 0.61 mmol), triethylamine, and bromoacetyl bromide (0.12 g, 0.61 mmol) were dissolved in dry dichloromethane, followed by stirring overnight at room temperature. The progress of the raw material reaction was monitored by TLC. The reaction solution was diluted with dried dichloromethane, then washed successively with saturated Na_2CO_3 and NaCl solutions. The organic layer was dried with anhydrous $MgSO_4$ and concentrated. The residue was purified with flash chromatography (methylene chloride:methanol = 20:1) to yield a solid white substance (0.09 g, 33.0%). ^{1}H NMR (600 MHz, DMSO-d_6) δ 8.35 (t, J = 5.6 Hz, 1H), 7.82 (dd, J = 8.8, 1.8 Hz, 2H), 6.98 (dd, J = 9.0, 2.9 Hz, 2H), 3.80 (d, J = 3.4 Hz, 3H), 3.54–3.46 (m, 7H), 3.39 (dq, J = 12.2, 5.8 Hz, 3H), 3.26–3.19 (m, 1H). MS m/z: [M+H]$^-$: 447.11. ^{13}C NMR (151 MHz, DMSO-d_6) δ 166.54, 166.20, 161.95, 129.44, 127.10, 113.90, 70.19, 70.09, 70.07, 69.46, 69.19, 55.78, 39.9, 40.3, 29.92.

Compound 6: 2-{2-[2-(2-hydroxyethoxy)ethoxy]ethoxy}ethyl-4-methoxybenzoate

Tetraethylene glycol (3.42 g, 17.59 mmol) and pyridine (0.5 ml) were dissolved in dried dichloromethane. Under nitrogen protection in an ice bath, p-methoxybenzoyl chloride (1 g, 5.86 mmol) was added drop by drop. The mixture was stirred at 0°C for 1 h and room temperature for 2 h. The progress of the raw material reaction was monitored by TLC. The mixture was extracted with ethyl acetate, then washed three times with distilled water and saturated NaCl solution. The organic layer was dried with anhydrous $MgSO_4$ and concentrated. The residue was purified with flash chromatography (dichloromethane:methanol = 20:1) to yield a colorless, transparent oily substance (2.32 g, 40.2%). ^{1}H NMR (600 MHz, DMSO-d_6) δ 7.95–7.89 (m, 2H), 7.08–7.03 (m, 2H), 4.57 (t, J = 5.5 Hz, 1H), 4.37–4.32 (m, 2H), 3.84 (s, 3H), 3.76–3.71 (m, 2H), 3.59 (dd, J = 6.0, 3.6 Hz, 2H), 3.55–3.44 (m, 8H), 3.42–3.37 (m, 2H). MS m/z [M+H]- :329.16, [M+Na]$^-$:351.16. ^{13}C NMR (151 MHz, DMSO-d_6) δ 165.86, 163.65, 131.75, 122.35, 114.51, 72.80, 70.35, 70.30, 70.23, 68.87, 64.19, 60.66, 55.99.

Compound 7: 14-bromo-13-oxo-3,6,9,12-tetraoxatetradecyl-4-methoxybenzoate (Z-X-2)

Compound 6 (0.6 g, 1.83 mmol), triethylamine (0.19 g, 1.83 mmol), and bromoacetyl bromide (0.37 g, 1.83 mmol) were dissolved in dry dichloromethane, followed by stirring overnight at room temperature. The progress of the raw material reaction was monitored by TLC. The reaction solution was diluted with dried dichloromethane, then the compound 7 was washed successively with saturated Na_2CO_3 and NaCl solutions. The organic layer was dried with anhydrous $MgSO_4$ and concentrated. The residue was purified with flash chromatography (petroleum ether:ethyl acetate = 1:1) to yield a colorless, transparent oily substance (0.35 g, 42.7%). ^{1}H NMR (600 MHz, DMSO-d$_6$) δ 7.94–7.89 (m, 2H), 7.08–7.03 (m, 2H), 4.37–4.32 (m, 2H), 4.23–4.17 (m, 2H), 4.16 (s, 2H), 3.84 (s, 3H), 3.75–3.71 (m, 2H), 3.59 (ddd, J = 9.7, 5.8, 3.8 Hz, 4H), 3.55–3.52 (m, 2H), 3.52 (s, 4H). MS m/z [M+H+Na]$^-$:473.06. ^{13}C NMR (151 MHz, DMSO-d$_6$) δ 168.97, 165.85, 163.65, 131.75, 122.35, 114.51, 72.80, 70.35, 70.30, 70.25, 70.23, 68.88, 65.42, 64.19, 55.99, 28.50.

2. Supporting results and experimental raw data

2.11· H NMR, ^{13}C NMR and MS spectra of the Z-X-1 and Z-X-2 compounds.

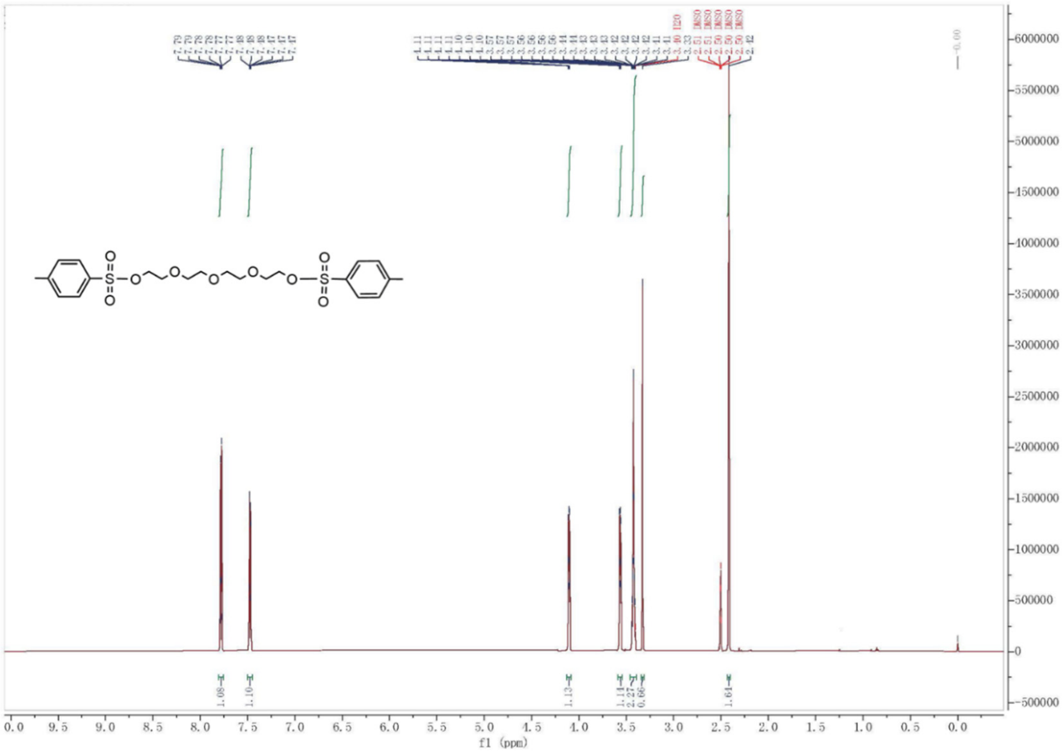

Figure S1. The ^{1}H NMR spectrum (600 MHz, DMSO) of compound 1.

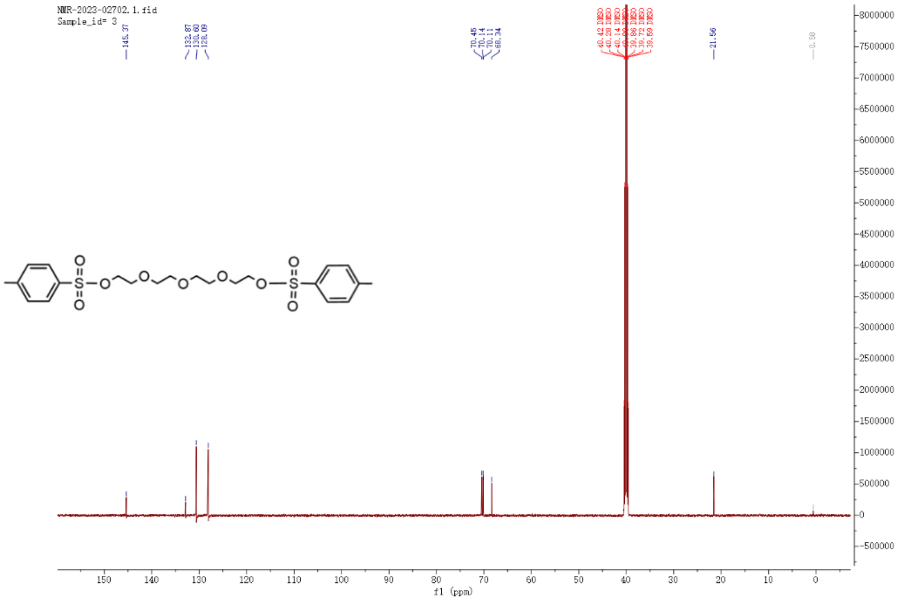

Figure S2. The ^{13}C NMR spectrum (151 MHz, DMSO-d_6) of compound 1.

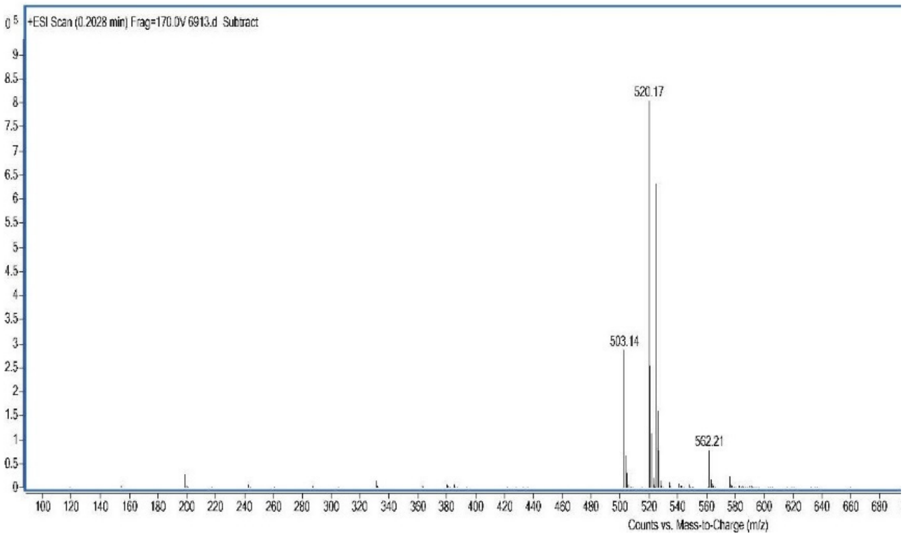

Figure S3. The MS spectra of compound 1.

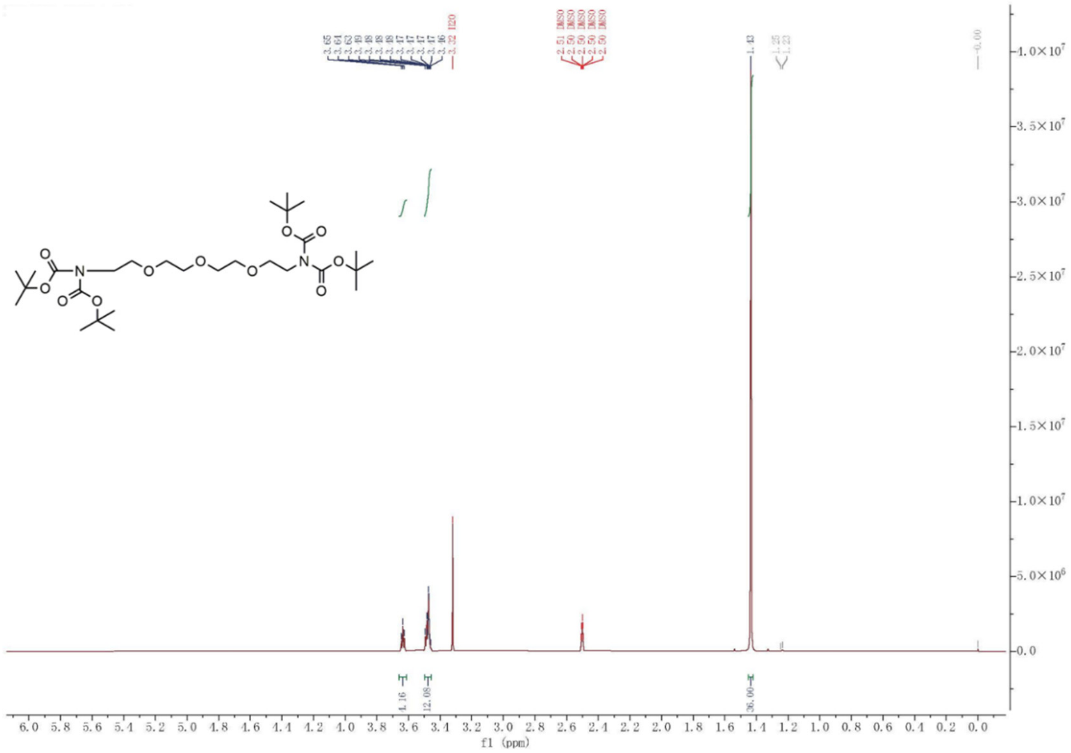

Figure S4. The ^{1}H NMR spectrum (600 MHz, DMSO) of compound 2.

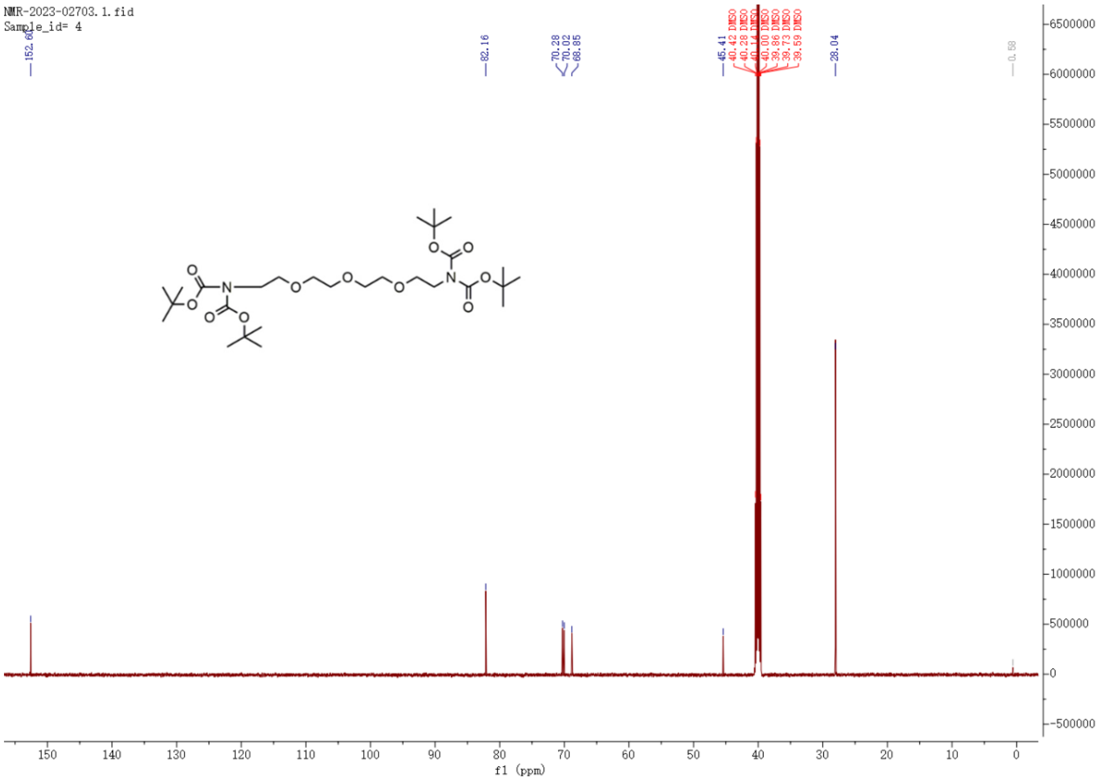

Figure S5. The ^{13}C NMR spectrum (151 MHz, DMSO-d_6) of compound 2.

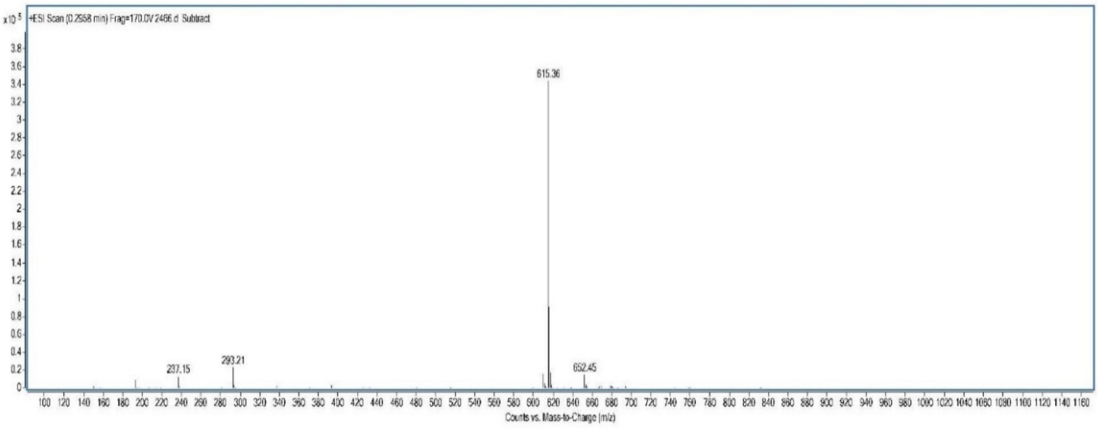

Figure S6. The MS spectra of compound 2.

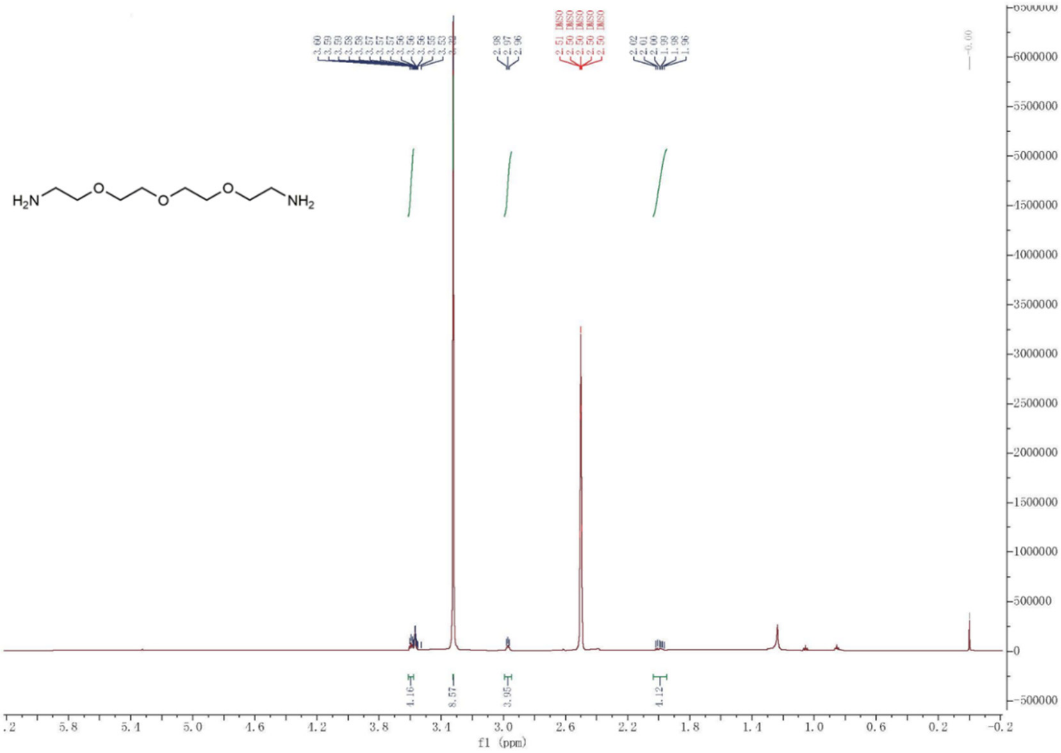

Figure S7. The ^{1}H NMR spectrum (600 MHz, DMSO) of compound 3.

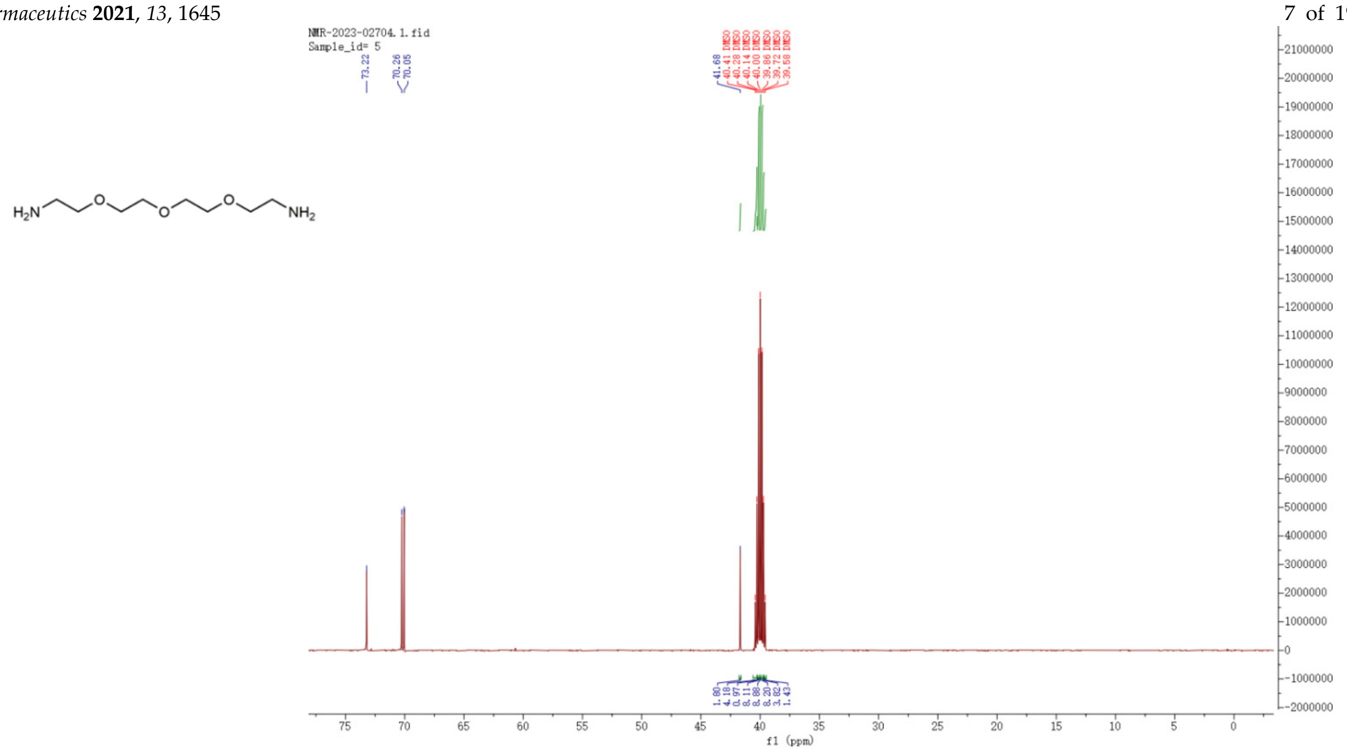

Figure S8. The ^{13}C NMR spectrum (151 MHz, DMSO-d_6) of compound 3.

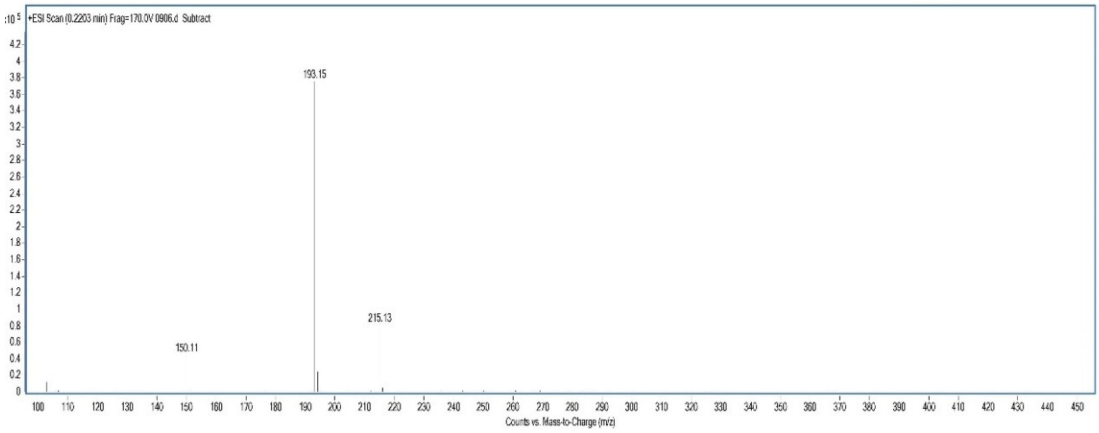

Figure S9. The HRMS spectra of compound 3.

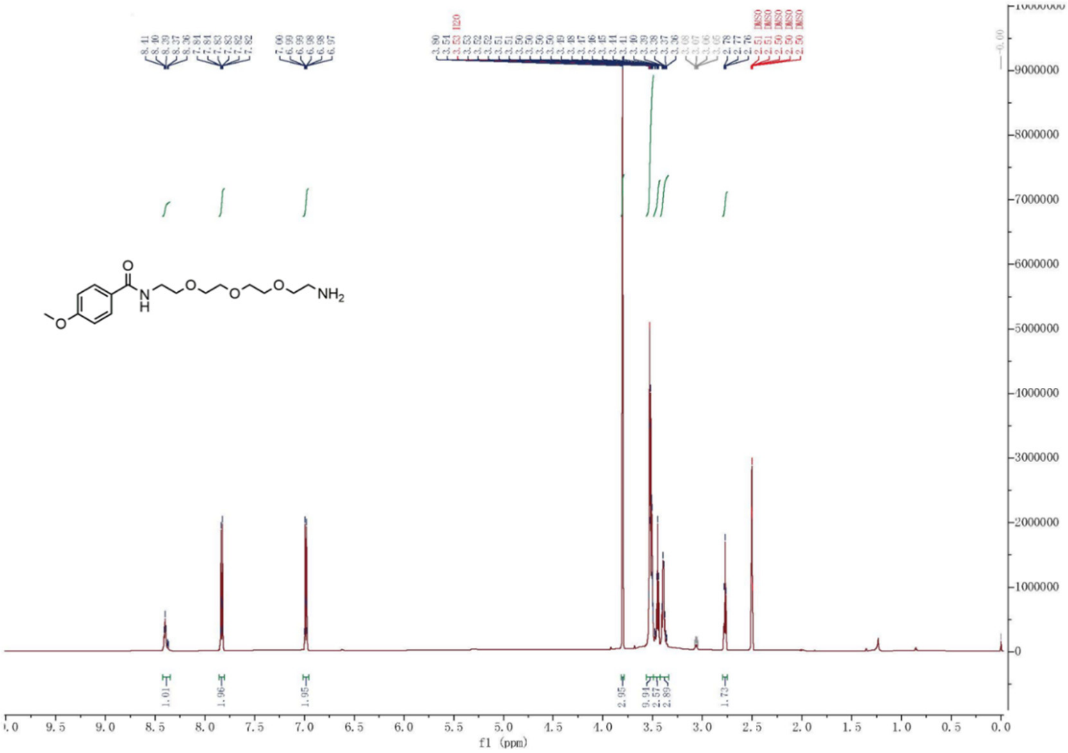

Figure S10. The ^{1}H NMR spectrum (600 MHz, DMSO) of compound 4.

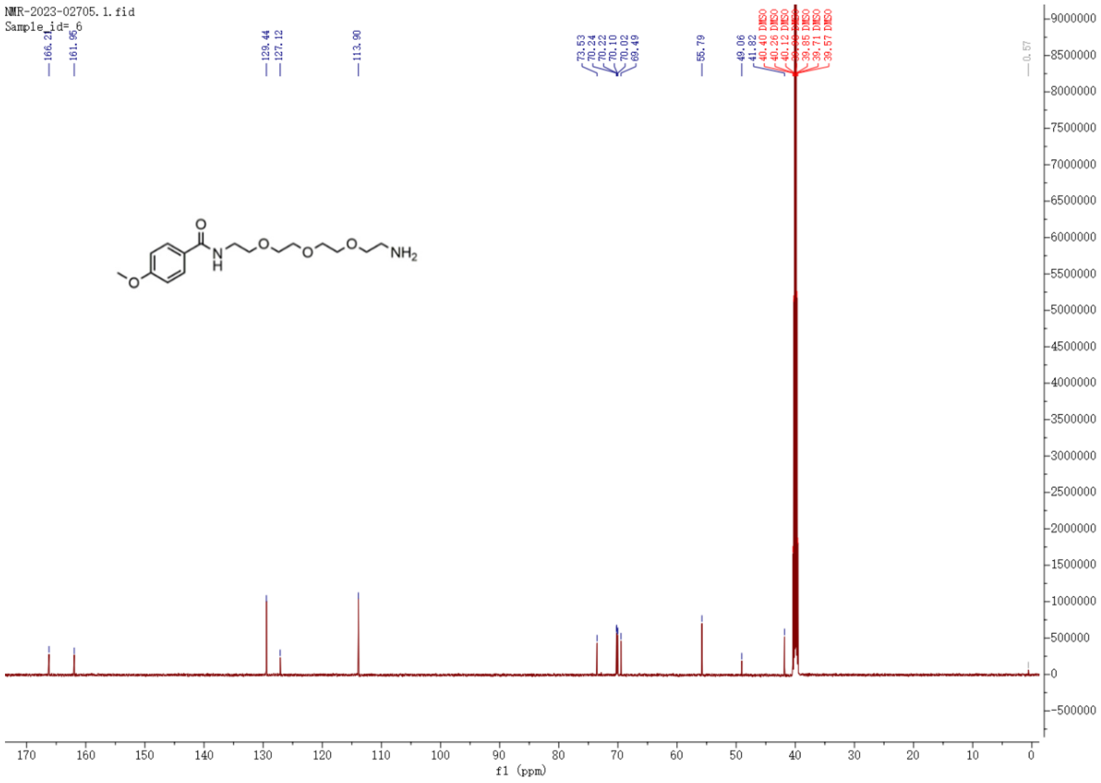

Figure S11. The ^{13}C NMR spectrum (151 MHz, DMSO-d_6) of compound 4.

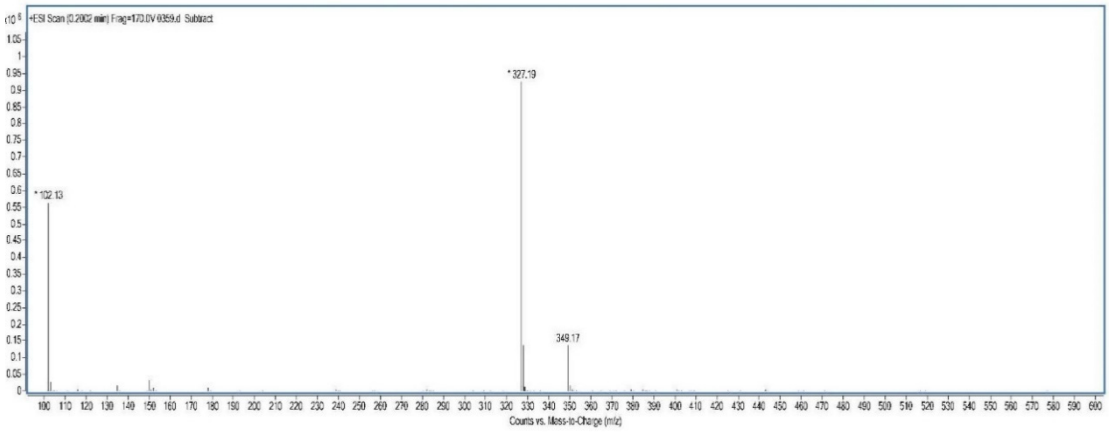

Figure S12. The MS spectra of compound 4.

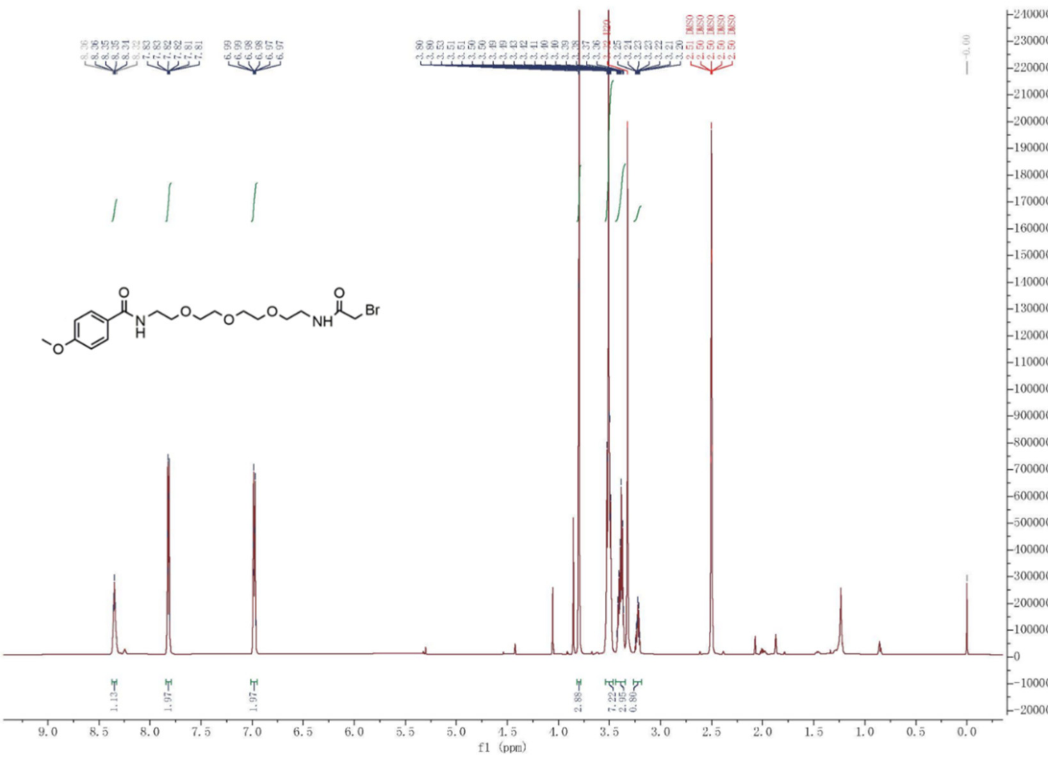

Figure S13. The ^{1}H NMR spectrum (600 MHz, DMSO) of compound 5.

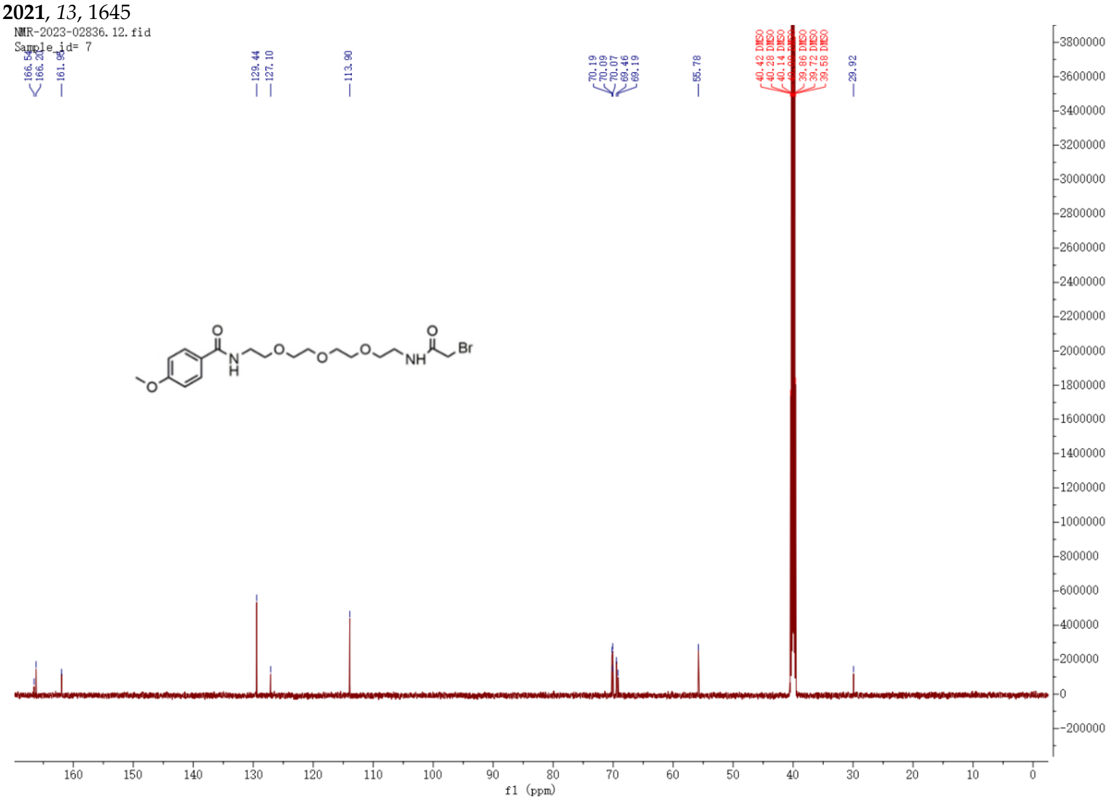

Figure S14. The ^{13}C NMR spectrum (151 MHz, DMSO-d$_6$) of compound 5.

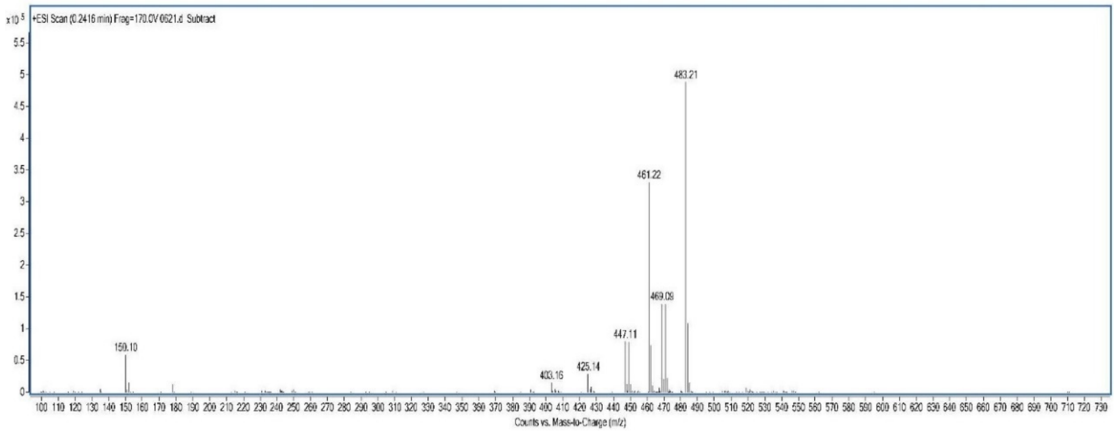

Figure S15. The MS spectra of compound 5.

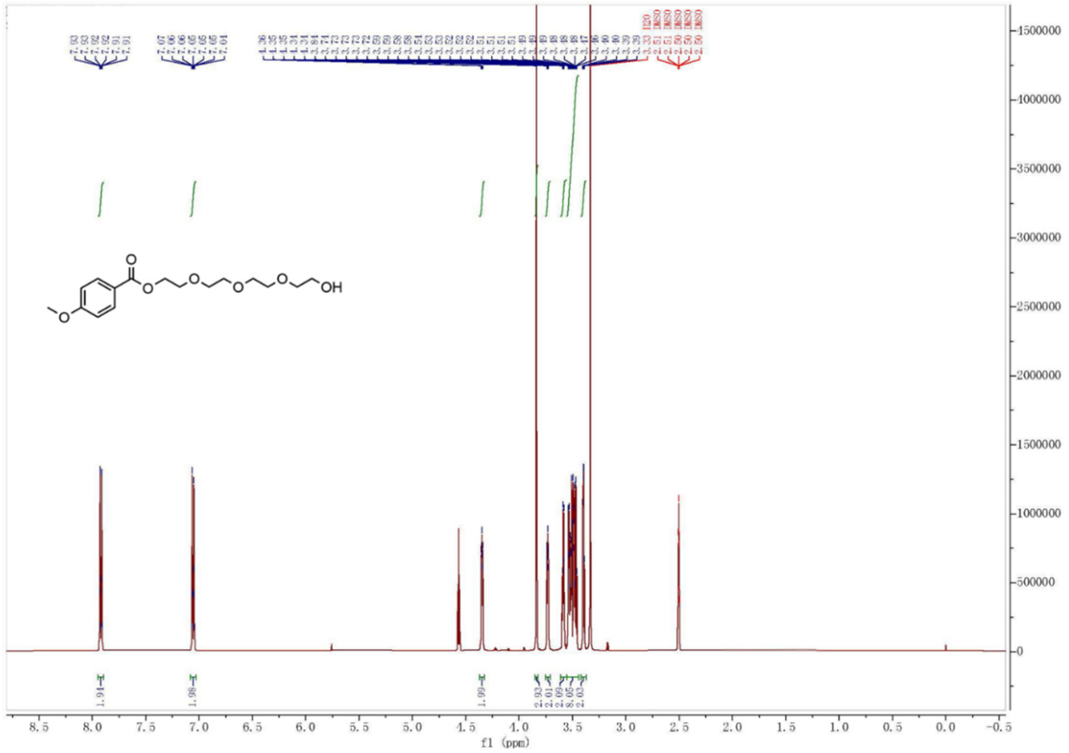

Figure S16. The ^{1}H NMR spectrum (600 MHz, DMSO) of compound 6.

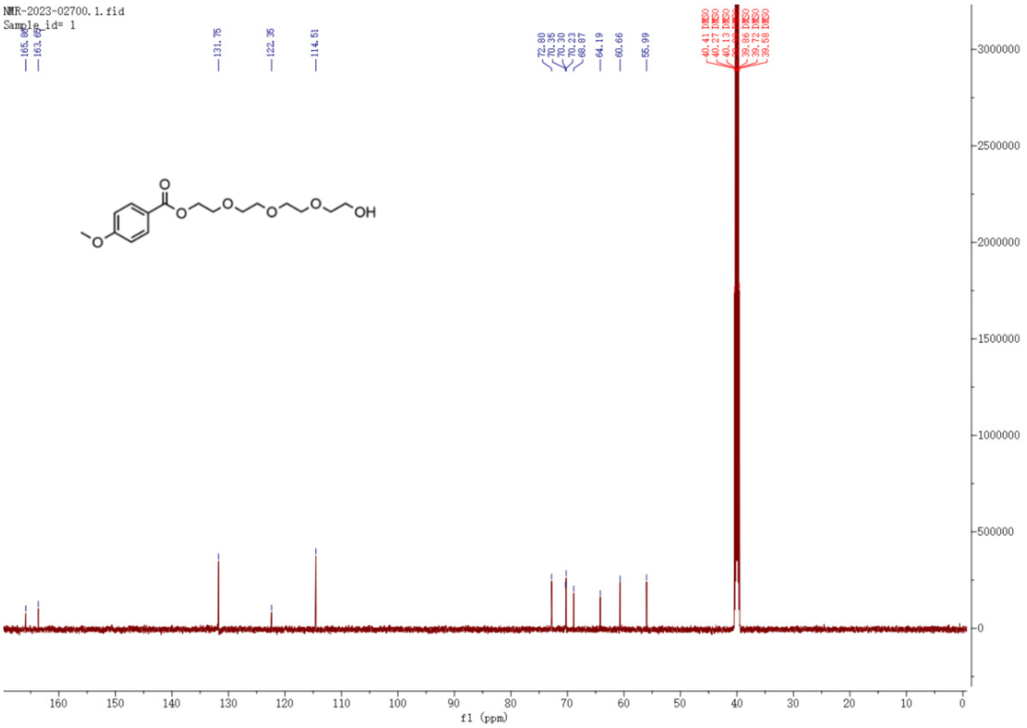

Figure S17. The ^{13}C NMR spectrum (151 MHz, DMSO-d_6) of compound 6.

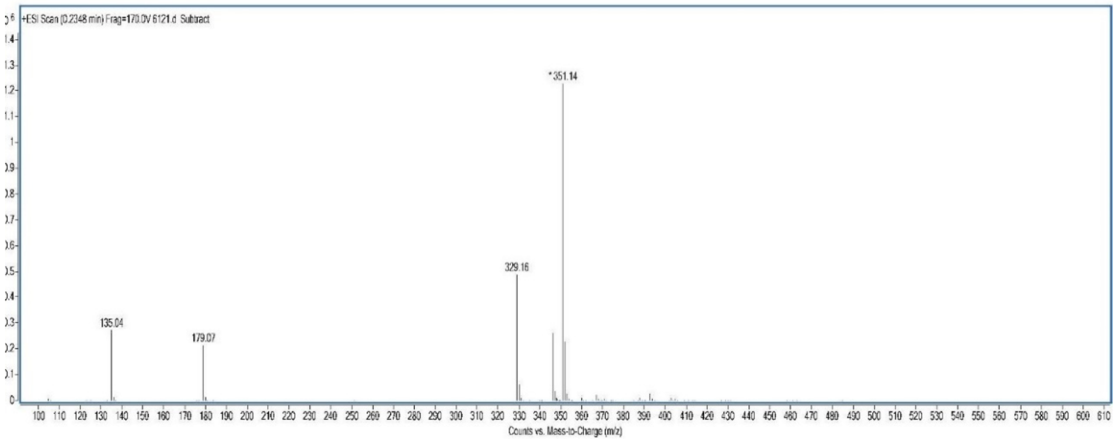

Figure S18. The MS spectra of compound 6.

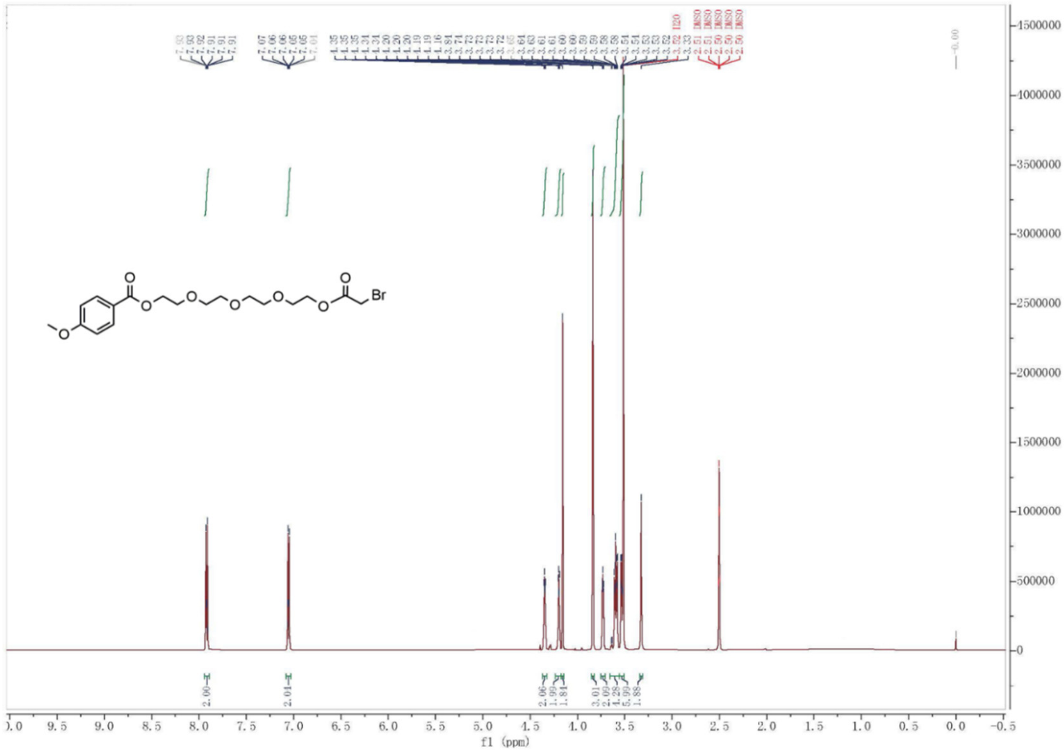

Figure S19. The ^{1}H NMR spectrum (600 MHz, DMSO) of compound 7.

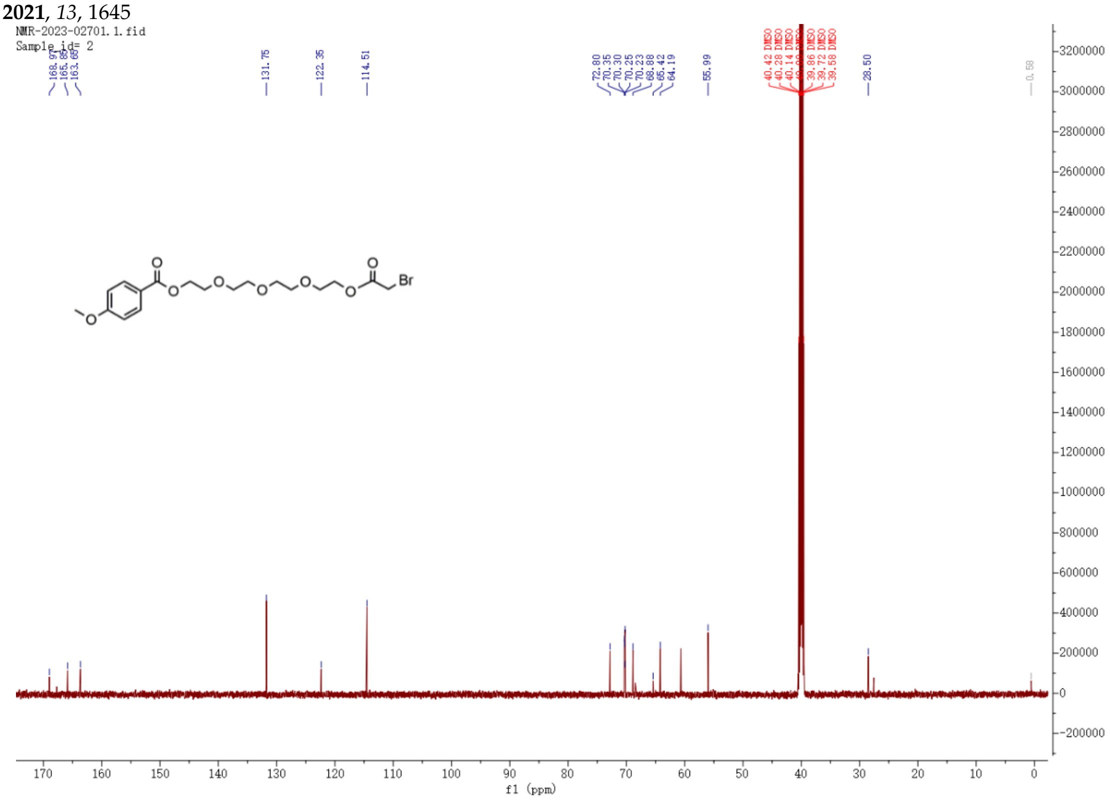

Figure S20. The ^{13}C NMR spectrum (151 MHz, DMSO-d$_6$) of compound 7.

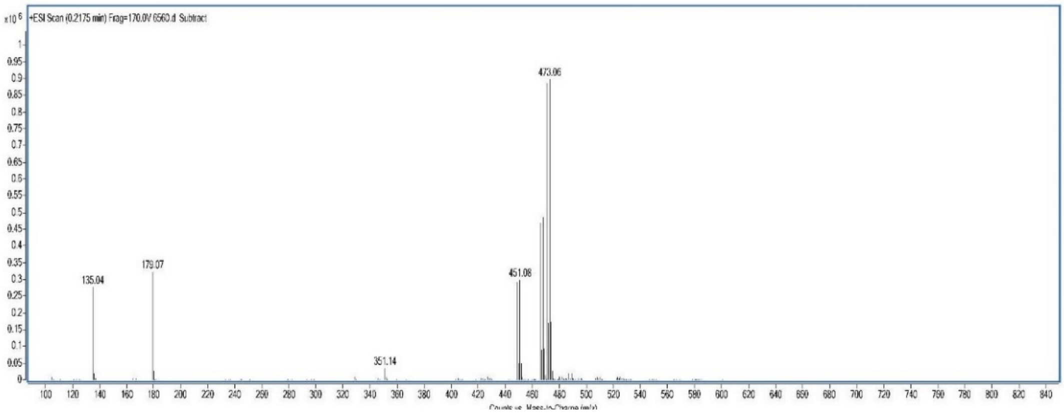

Figure S21. The MS spectra of compound 7.

2.2. The sequences and molecular weights of Z1-Z4

Table S1. the sequences and molecular weights of Z1-Z4.

No.	Sequence (5′-3′)	Molecular weight (estimated)	Molecular weight (actual)
Z1	GGCTAAATCGCTCCAC-CAA*G	6088.0	6087.6
Z2	G*GCTAAATCGCTCCAC-CAAG	6088.0	6087.6
Z3	GGCTAAATCG*CTCCAC-CAAG	6088.0	6087.6
Z4	G*GCTAAATCGCTCCAC-CAA*G	6195.8	6196.8

* represented PS modification.

2.3. The MALDI-TOF-MS spectra of Z1-Z4.

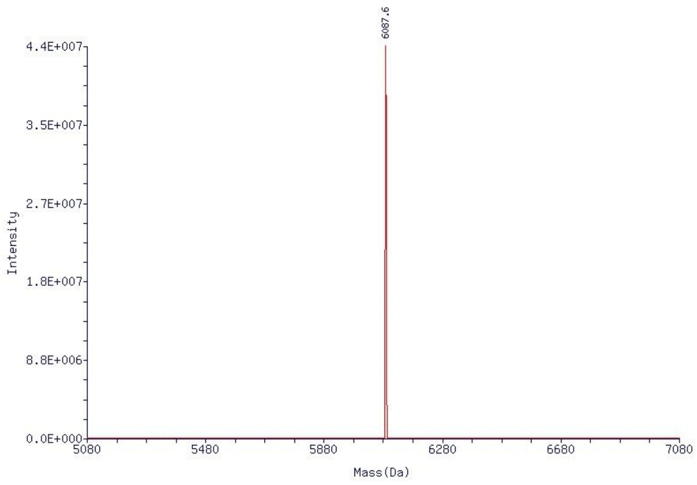

Figure S22. The MALDI-TOF-MS of Z1.

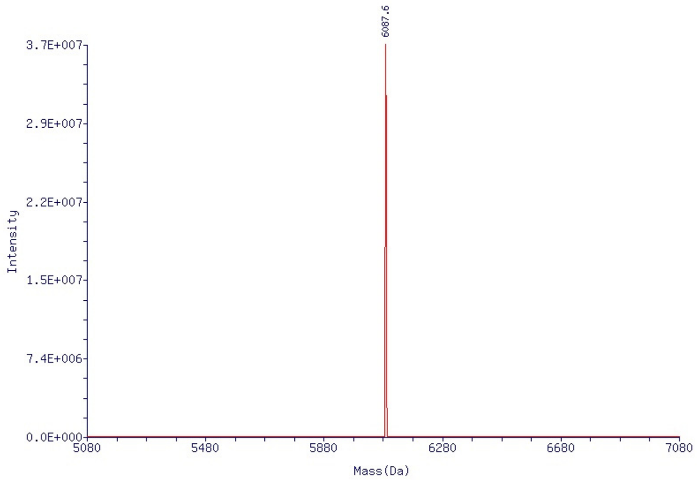

Figure S23. The MALDI-TOF-MS of Z2.

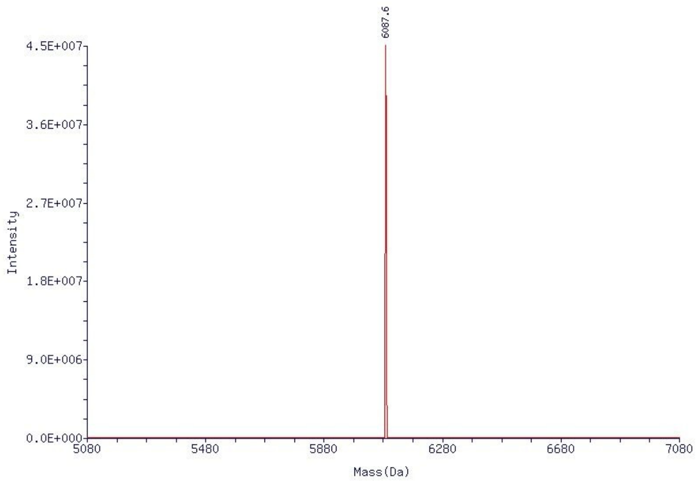

Figure S24. The MALDI-TOF-MS of Z3.

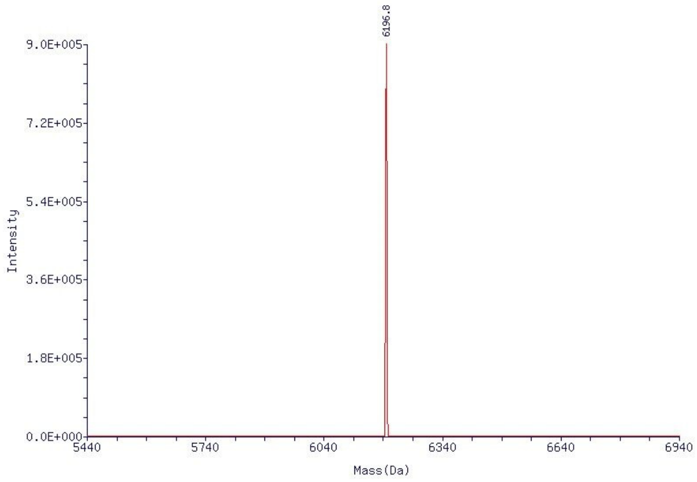

Figure S25. The MALDI-TOF-MS of Z4.

2.4. The MALDI-TOF-MS and HPLC spectra of T1-T6.

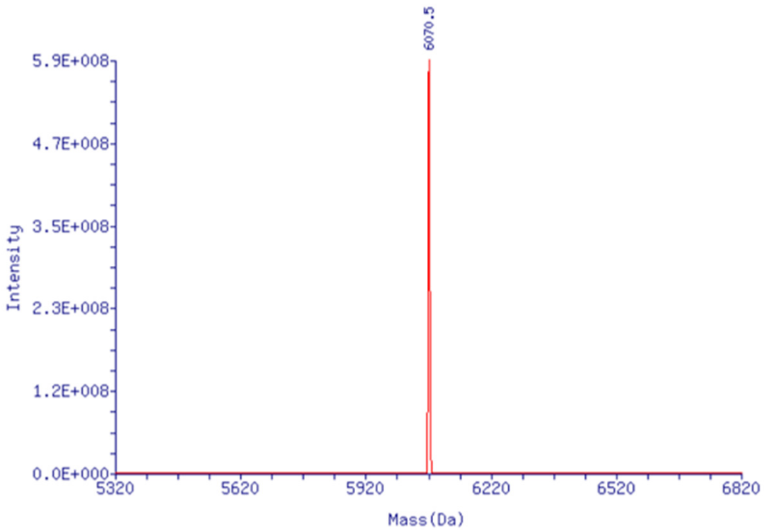

Figure S26. The MALDI-TOF-MS of T1.

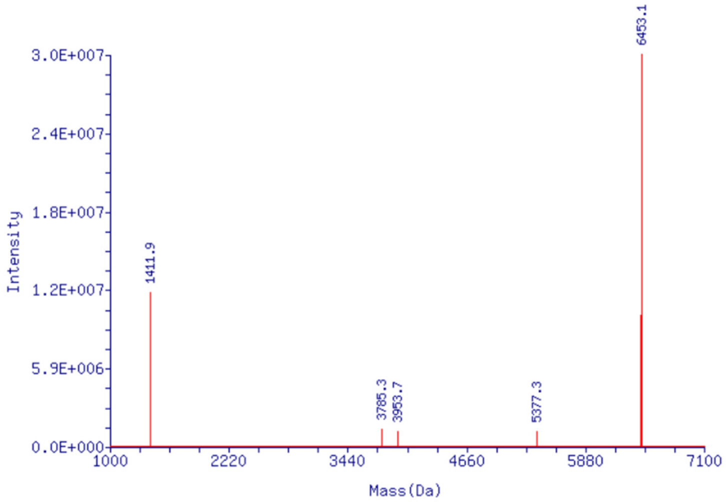

Figure S27. The MALDI-TOF-MS of T2.

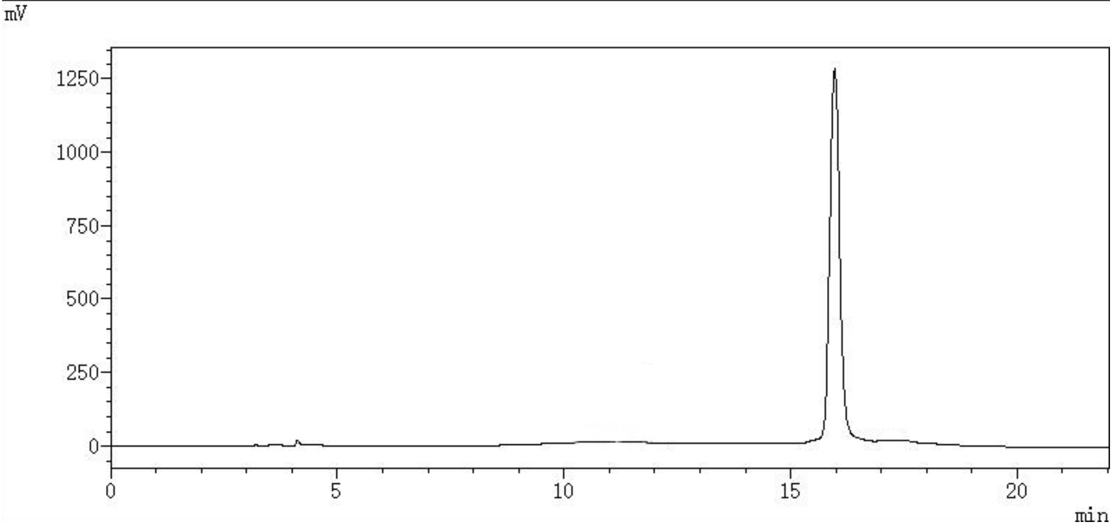

Figure S28. The HPLC spectra of T2.

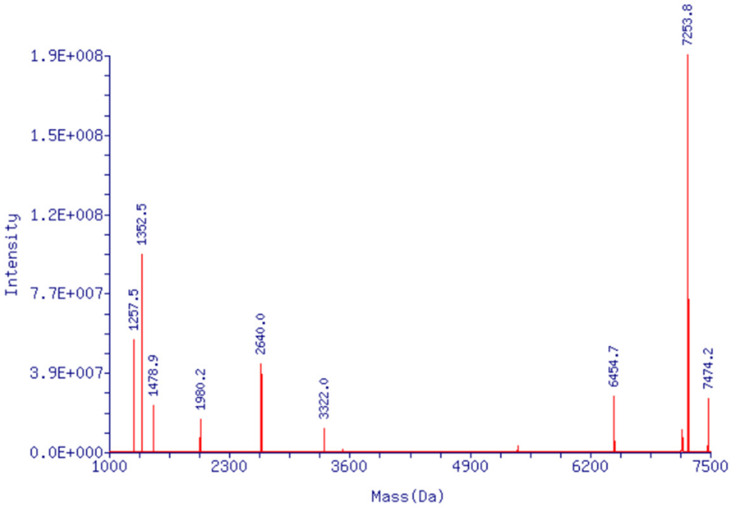

Figure S29. The MALDI-TOF-MS of T3.

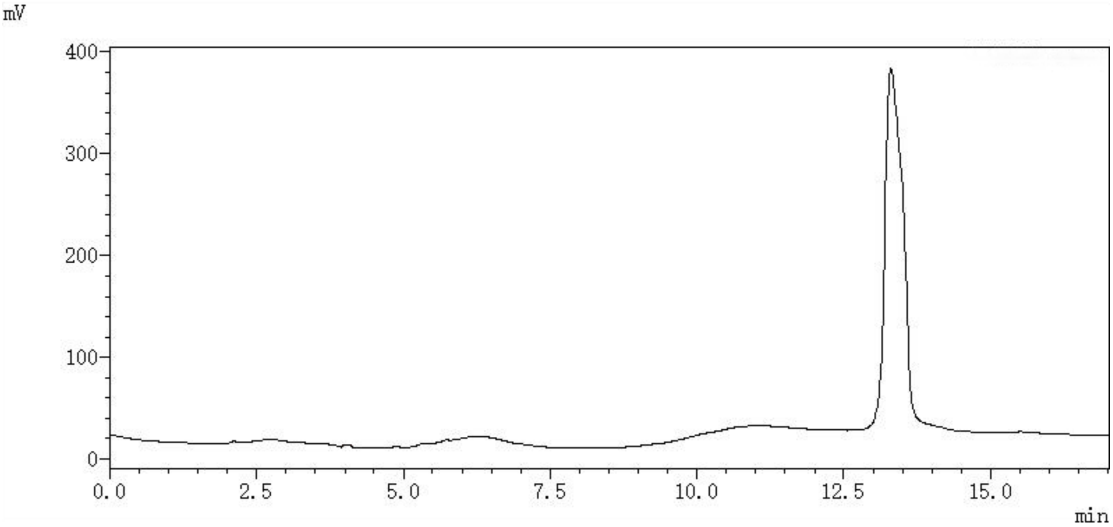

Figure S30. The HPLC spectra of T3.

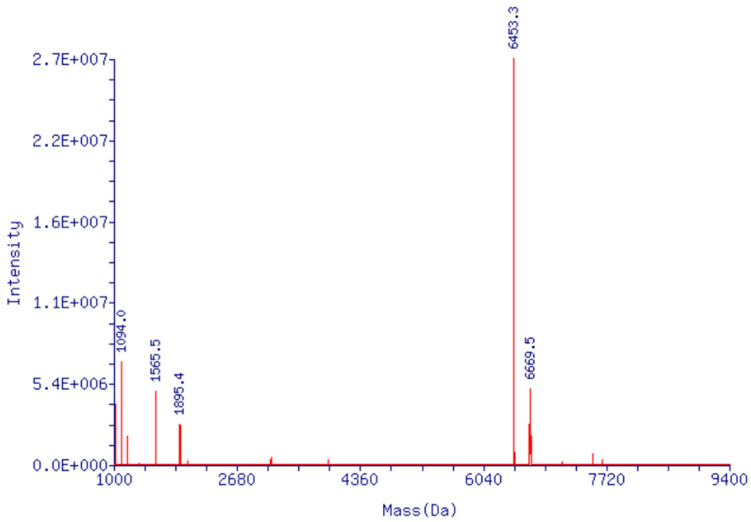

Figure S31. The MALDI-TOF-MS of T4.

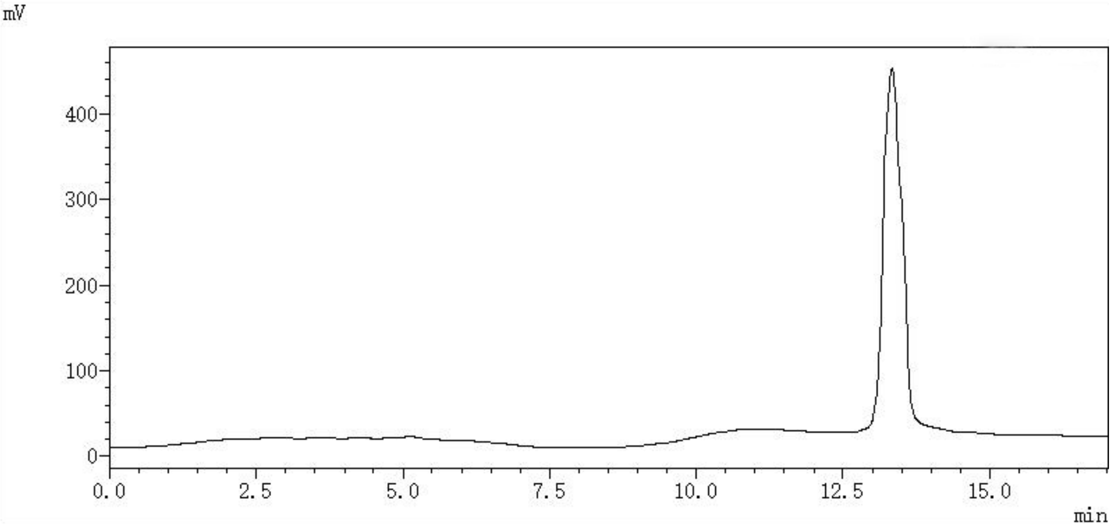

Figure S32. The HPLC spectra of T4.

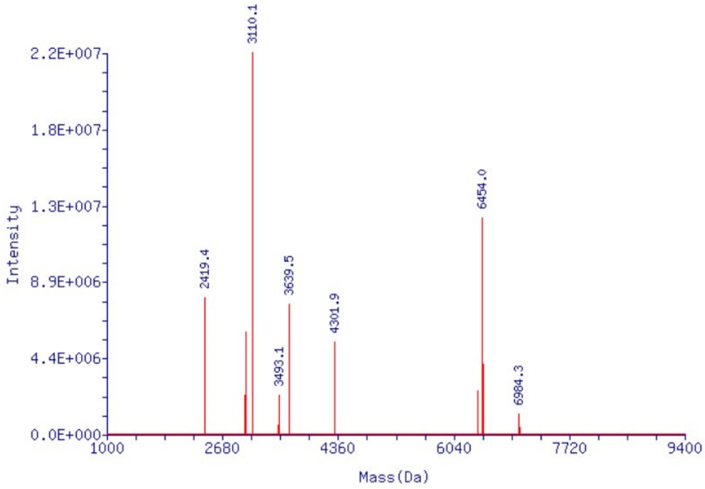

Figure S33. The MALDI-TOF-MS of T5.

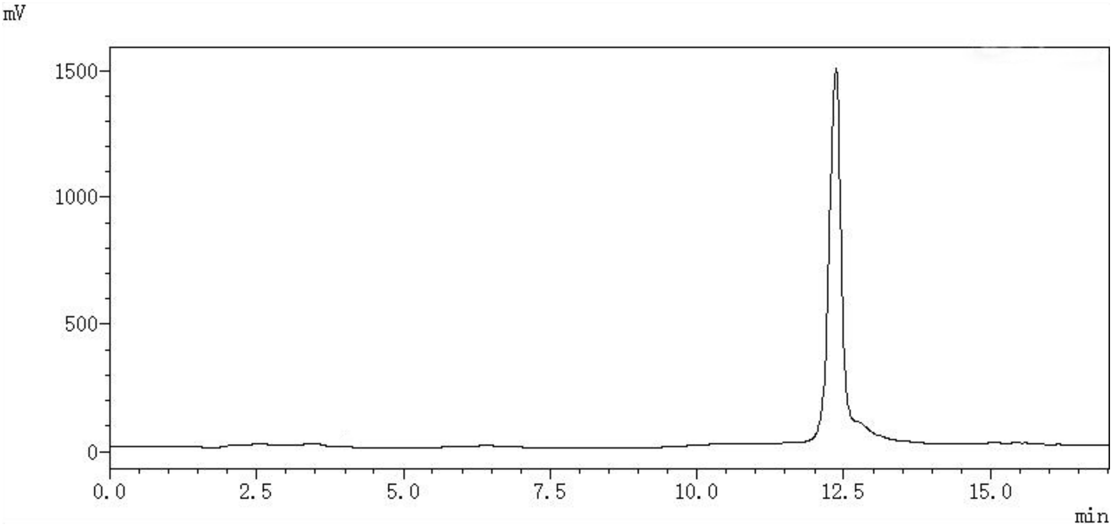

Figure S34. The HPLC spectra of T5.

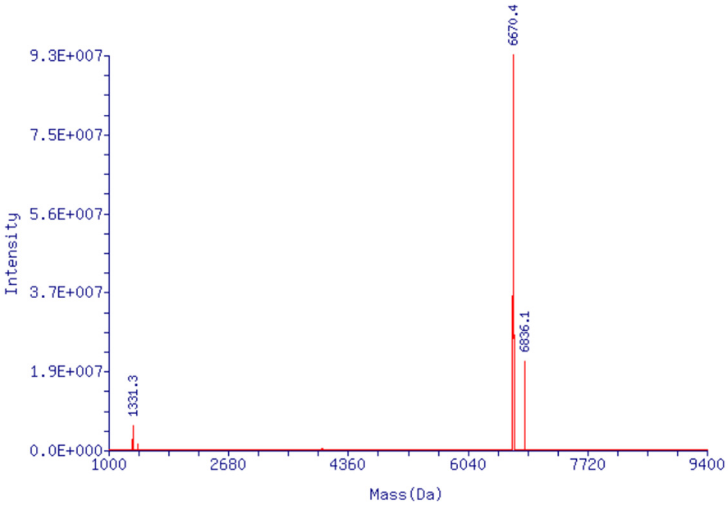

Figure S35. The MALDI-TOF-MS of T6.

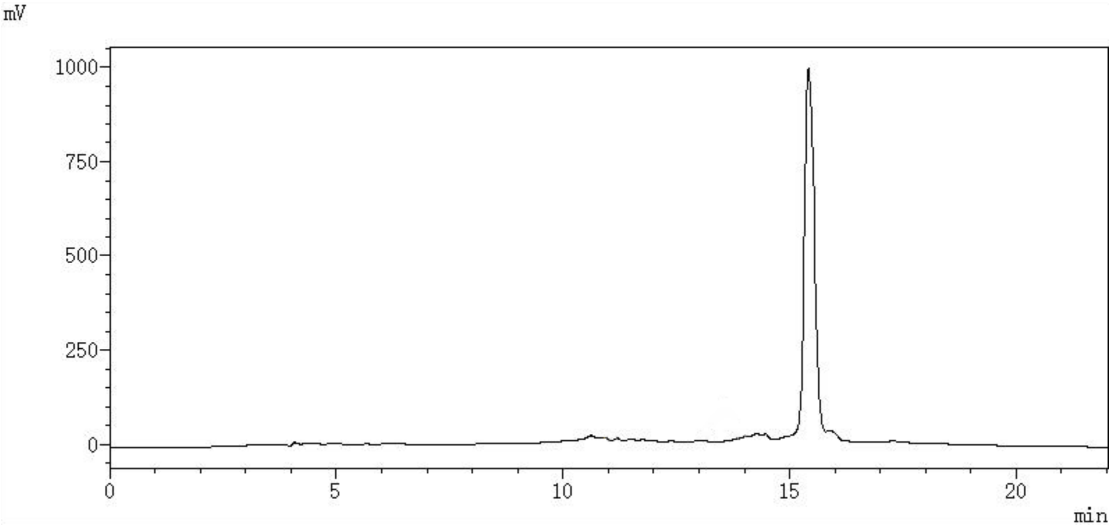

Figure S36. The HPLC spectra of T6.

2.5. The cellular uptake ability of T1–T3.

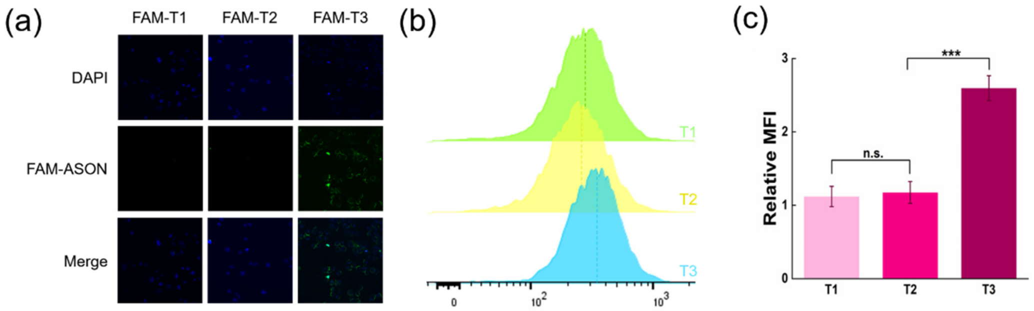

Figure S37. The cellular uptake ability of T1, T2 and T3. (a) Confocal images and (b) flow cytometry analysis of MCF-7 cells treated with PBS and T1–T3 [nuclei were labeled with DAPI (blue) and oligonucleotides was labeled with FAM (green); scale bars = 40 μm]. (c) Relative MFIs of treated MCF-7 cells. n.s., $p > 0.05$; ***$p < 0.001$. The data are presented as means ± standard deviations ($n = 3$).
